# Discovery of andrographolide hit analog as a potent cyclooxygenase-2 inhibitor through consensus MD-simulation, electrostatic potential energy simulation and ligand efficiency metrics

**DOI:** 10.1038/s41598-023-35192-7

**Published:** 2023-05-19

**Authors:** Priyanka Jain, Jitendra Satija, C. Sudandiradoss

**Affiliations:** 1 School of Biosciences and Technology, Vellore Institute of Technology, Vellore, Tamil Nadu 632014 India; 2 Centre for Nanobiotechnology, Vellore Institute of Technology, Vellore, Tamil Nadu 632014 India

**Keywords:** Virtual drug screening, Molecular modelling, Cheminformatics, Bioinformatics

## Abstract

Cyclooxygenase-2 (COX-2) is the key enzyme responsible for the conversion of arachidonic acid to prostaglandins that display pro-inflammatory properties and thus, it is a potential target protein to develop anti-inflammatory drugs. In this study, chemical and bio-informatics approaches have been employed to find a novel potent andrographolide (AGP) analog as a COX-2 inhibitor having better pharmacological properties than aspirin and rofecoxib (controls). The full amino acid sequenced human Alpha fold (AF) COX-2 protein (604AA) was selected and validated for its accuracy against the reported COX-2 protein structures (PDB ID: 5F19, 5KIR, 5F1A, 5IKQ and 1V0X) followed by multiple sequence alignment analysis to establish the sequence conservation. The systematic virtual screening of 237 AGP analogs against AF-COX-2 protein yielded 22 lead compounds based on the binding energy score (< − 8.0 kcal/mol). These were further screened out to 7 analogs by molecular docking analysis and investigated further for ADMET prediction, ligand efficiency metrics calculations, quantum mechanical analysis, MD simulation, electrostatic potential energy (EPE) docking simulation, and MM/GBSA. In-depth analysis revealed that AGP analog A3 (3-[2-[(1R,4aR,5R,6R,8aR)-6-hydroxy-5,6,8a-trimethyl-2-methylidene-3,4,4a,5,7,8-hexahydro-1H-naphthalen-1-yl]ethylidene]-4-hydroxyoxolan-2-one) forms the most stable complex with the AF-COX-2 showing the least RMSD value (0.37 ± 0.03 nm), a good number of hydrogen bonds (protein–ligand H-bond = 11, and protein H-bond = 525), minimum EPE score (− 53.81 kcal/mol), and lowest MM-GBSA before and after simulation (− 55.37 and − 56.25 kcal/mol, respectively) value compared to other analogs and controls. Thus, we suggest that the identified A3 AGP analog could be developed as a promising plant-based anti-inflammatory drug by inhibiting COX-2.

## Introduction

Cyclooxygenase (COX or prostaglandin G/H synthase) plays a vital role in the generation of biological mediators such as prostaglandins (PGs), thromboxane, and prostacylins from arachidonic acid. The COX enzyme exists as two isoforms: (i) the COX-1 isozyme is constitutively active and primarily involved in homeostatic functions like the regulation of platelet aggregation and gastric acidity; (ii) in contrast, the COX-2 isomeric form induces during pathological conditions such as inflammation, pain, and fever^1–3^. To develop anti-inflammatory and analgesic drugs, COX-2 is a viable and specific target and various COX-2 inhibitory drugs have been developed in the past few decades^4^. The COX-2 comprises 604 amino acid sequence length with three major domains: (i) the epidermal growth factor (EGF) domain (34–72 amino acid), (ii) the membrane binding domain (73–116 amino acid), and (iii) the catalytic domain which contains the COX and peroxidase active sites [UniProt- P35354]. The COX and peroxidase active sites are spatially distinct but functionally linked^5^. The proximity to Tyr371 and Ser516 are the critical catalytic amino acids for the COX-2 active site^6^. The valine residue at the 509 position in the COX-2 forms a hydrophobic (HYD) pocket at its active site and plays a key role in the selective induction of the enzyme and thus inflammatory response^7,8^.

The COX-2 catalyzes the reaction in two steps: (i) the first step is the COX reaction in which arachidonate is converted to PG-G2 which occurs in the HYD channel of the core of the protein and (ii) the second step is the peroxidase reaction wherein PG-G2 is reduced to PG-H2 (an important precursor of prostacyclin and expressed during inflammation) that takes place at the heme-containing active site located near the protein surface^9^. Non-steroidal anti-inflammatory drugs (NSAIDs), especially coxibs, and aspirin, reduce inflammation by inhibiting the PG synthesis via covalent interaction with Ser-516 residue at the COX-2 active site^10,11^. Although these drugs are safe and effective, they can cause gastrointestinal toxicity, which has limited their use in highly sensitive patients. Designing of selective COX-2 inhibitors including celecoxib and rofecoxib (coxibs), inhibit COX-2 instead of COX-1 in order to provide pain relief and anti-inflammatory properties and prevent the gastric discomforts experienced with non-selective NSAIDs^12–14^. Rofecoxib is a highly selective NSAID due to the absence of carboxylic acid (thus less GI irritant) and the presence of two large aromatic cycles linked to a central heterocycle ring^15^. Beside the selectivity and efficacy, the high cost of the drug, potential adverse cardiovascular effects, and the increased risk of ischemic stroke, use of COX-2 inhibitors is controversial^16–18^. Hence, there is an urgent demand for a therapeutic drug against COX-2 with minimal or no side effects preferably from the natural origin^19^.

Secondary metabolites such as alkaloids, terpenoids, stilbenes, flavonoids, saponins, and fatty acids have been demonstrated to have COX-2 inhibitory activity^20^. Amongst these, andrographolide (AGP), which is obtained from the *Andrographis paniculata* plant, has been proven as an important natural anti-inflammatory agent along with various other pharmacological benefits^21–23^. Andrographolide is a natural electrophilic covalent inhibitor, derived from the terpenoid pathway and privileged with the structural features which are necessary for binding with multiple targets^24–26^. Structurally, AGP contains a complex 3D architecture with a high number of sp^3^-hybridized carbons, rich oxygen content, low nitrogen content, a polycyclic structure with aliphatic side chains, and no aromatic rings. Moreover, the chemical properties of andrographolide are strengthened by α-Alkylidene-β-hydroxy-γ-butyrolactone moiety which is a specific electrophilic fragment that enables it to irreversibly and covalently modify the target protein with thiol-containing amino acid residue^25^. This provides substantially to the binding affinity with the target protein and presents andrographolide as an efficient covalent inhibitor^26,27^.

A few research groups have recently attempted to understand the andrographolide signaling pathway targets which are directly or indirectly involved in the inflammatory reaction^28^. Tran et al*.* (2020) reported the anti-inflammatory activity of the andrographolide and its few derivatives in a lipopolysaccharide-induced acute lung injury model and emphasized that α–Alkylidene-β-hydroxy-γ-butyrolactone and ent-labdane moieties are vital for anti-inflammatory activity^25^. Dai et al*.* (2011) reported that AGP exerts its enhanced anti-inflammatory effect by decreasing serum inducible nitric oxide synthase (iNOS) activity, NO production, and PGE2 production^29^. Recently, Wang et al*.* (2019) reported that AGP and its derivative effectively inhibit the lipopolysaccharide-induced expression of COX-2 in murine macrophages^30^. Similarly, other research groups have reported that the andrographolide demonstrates anticancer activity by inhibiting the expression of COX-2 protein^31,32^. Although efforts have been paid to prove the anti-inflammatory activity of andrographolide, the pharmacokinetic parameters, i.e. poor solubility, and low bioavailability are the limiting factors for its successful drug development^33^.

In this study, systematic computational analysis is carried out to find out a novel natural plant-based andrographolide analog as an anti-inflammatory agent against COX-2 enzyme which has better potency, efficacy, and selectivity than aspirin and rofecoxib (controls). Molecular mechanics techniques of cheminformatics and quantum mechanical approaches are employed for detailed investigation. The full sequence length of the human AF-COX-2 protein structure is used after the validation against the available 3D crystal structure. Further, multiple sequence alignment was performed for the amino acid sequence length of the protein to find out conserved regions for biological activity. A total of 237 AGP analogs are used for screening followed by docking studies for the hit analogs, ADMET parameters prediction, ligand efficiency metrics calculations, in-depth quantum mechanical analysis, and MM/GBSA rescoring studies. Further, the stability of the hit AGP analogs complex was assessed by the molecular docking simulation and electrostatic potential energy (EPE) docking simulation analysis. Outcomes of the present study help in the development of plant-based COX-2 inhibitor as a future anti-inflammatory candidates.

## Materials and methods

### Selection, validation, refinement, and binding site prediction of COX-2 protein

The 3D structure of crystallized COX-2 (5F19, 5KIR, 5F1A, 5IKQ, and 1V0X) protein was retrieved from the PDB database while the predicted structure of human COX-2 protein, containing 604 amino acids, was obtained from the AlphaFold (AF) (ID: AF-P35354-F1)^34–37^. The protein structure was validated by SAVES v6.0 (https://saves.mbi.ucla.edu/), ProSA, and ProQ^38–42^. For the accuracy of the AF-COX-2 human protein structure, the validation results were compared with other available crystallized COX-2 PDB structures, i.e. 5F19, 5KIR, 5F1A and 5IKQ, and predicted COX-2 model (1V0X). Before any interaction analysis, the AF-COX-2 protein structure was refined by 3Drefine (https://3drefine.mu.hekademeia.org/)^43^. Subsequently, possible binding pockets for the AF-COX-2 protein were predicted by CASTp 3.0 and visualized by Chimera^44,45^.

### Multiple sequence alignment

To verify the presence of the conserved amino acids in the AF-COX-2 protein, multiple sequence alignment (MSA) was performed using the PRALINE program^46^. For the same, 10 different mammal species namely *Homo sapiens, Rattus norvegicus, Mus musculus, Ovis aries, Bos Taurus, Oryctolagus cuniculus, Cavia porcellus, Equus caballus, Neovison vison, and Gallus gallus* were selected and amino acid sequences of their respective COX-2 protein were retrieved from Uniprot (http://www.uniprot.org/). The resulting aligned amino acid sequences were colored based on the conservation index (0 to10), wherein the highly conserved sequences of amino acid amongst species are responsible for the particular biological function of the protein^47^.

### Site-directed virtual screening and molecular docking analysis

A total of 237 AGP analogs were taken from the PubChem database that consists of the common α,β-unsaturated γ-lactone moiety which is responsible for the biological activity by forming adduct with the residues during the biological interaction^25,26^. Then, the analogs were converted into a autodock PDBQT format for virtual screening by Open Babel^48,49^. PyRx (AutoDock Vina) software was used for the site-directed virtual screening of the AGP analogs against the AF-COX-2 protein^50^. Further, based on the interacting amino acid residues present for the co-crystal aspirin and rofecoxib in the 5F19 and 5KIR PDB structure respectively, amino acids namely His75, Arg106, Val330, Tyr334, Val335, Tyr341, Tyr371, Trp373, Arg499, Phe504, Glu510, Ser516, and Leu517 were chosen as active site residues in the AF-COX-2 protein for setting up the grid for the virtual screening^35,36^. The grid box was set with search space center 4.76, 5.07, and − 0.12 at x, y, and z, respectively, with 8 exhaustiveness, while the dimensions (Å) were set for the grid at x, y, and z with a value of 29.54, 29.74, and 27.37 each. For shortlisting the hit AGP analogs, the cutoff value for binding energy (BE) was set to be lesser than − 8.00 kcal/mol.

The hit AGP analogs obtained from virtual screening were submitted for molecular docking analysis along with AGP, aspirin, and rofecoxib as controls. AutoDock4.2 was utilized for the site-directed docking of these compounds with AF-COX-2^51^. Since the AF protein structure of COX-2 is a predicted protein structure, co-crystal ligands, i.e. aspirin and rofecoxib of the existing crystallized structure of COX-2 protein 5F19 and 5KIR, respectively, were taken as reference compounds for the docking analysis. Further, amino acids i.e. His75, Arg106, Val330, Tyr334, Val335, Tyr341, Tyr371, Trp373, Arg499, Phe504, Glu510, Ser516, and Leu517 were chosen as the binding site residues based on the aspirin and rofecoxib interacting residues in the 5F19 and 5KIR PDB structures. The gird box for docking was generated with 0.375 Å spacing and dimension along the x, y, and z coordinates with the values of 84, 88, and 94, respectively. The grid center was set to the centroid of the binding site residues with the dimension of 4.305, 4.394, and 1.611, respectively, alongside the x, y, and z–Axis. Further, GA (genetic algorithm) was employed to calculate the docking parameters with 25000000 number of energy evaluations, 27000 number of generations, and 2.0 Å RMSC (root mean square cluster) tolerance. Lamarckian genetic algorithm was used to perform docking simulation. The AutoGrid and AutoDock were utilized to perform molecular docking and to investigate the best binding pose of the ligands in protein–ligand complexes. Finally, the ligand’s conformers with the lowest free binding energy were selected for further analysis. The output files obtained from the docking study were visualized and analyzed by ICM-Browser and Accelrys discovery studio visualizer^52^.

### ADMET prediction

ADMETlab 2.0 was used to predict physicochemical, medicinal, absorption, distribution, metabolism, excretion, and toxicological parameters for AGP and shortlisted AGP analogs along with aspirin and rofecoxib^53^. It was also used to establish the drug-likeness of the AGP and shortlisted analogs.

### Ligand efficiency metrics

Ligand binding efficiency metrics were computed in terms of inhibition constant (Ki), ligand efficiency (LE), ligand lipophilic efficiency (LLE), ligand efficiency scaling function (LE_Scale), fit quality (FQ), and lipophilicity corrected ligand efficiency (LELP) by following the Eq. (1–6)^54–58^.

1 $${\text{Ki }} = {\text{ exp}}\left( {{\text{Binding Energy}}/{\text{RT}}} \right)$$

2 $${\text{LE }} = \, - {\text{Binding Energy/Heavy Atom}}$$

3 $${\text{LLE }} = \, - {\text{logKi }}{-}{\text{ LogP}}$$

4 $${\text{LE}}\_{\text{Scale }} = 0.873{\text{e}}^{{ - 0.026 \times {\text{HA }}}} {-} \, 0.0{64}$$

5 $${\text{FQ }} = {\text{ LE }} \div {\text{ LE\_Scale}}$$

6 $${\text{LELP }} = {\text{ LogP }} \div {\text{ LE}}$$

### Quantum–mechanical descriptors calculation

Spartan 20 (wavefun.com) package was used to calculate quantum–mechanical (QM) descriptors in terms of the highest occupied molecular orbital (HOMO), lowest unoccupied molecular orbital (LUMO), HOMO–LUMO gap (HLG), and EPE parameters for assessing the molecular orbital properties, electrostatic potential, and elucidating various types of interactions. All the chemical compounds were optimized to compute equilibrium geometric variables in the gaseous state at the ground employing density functional theory (DFT) with Becke’s three-parameter exchange potential^59^. Further, Lee Yang Parr (LYP) correlation functional, which is an exchange functional combining of Becke with a 6-31G* basis set, was used to calculate the ground state geometries^60–62^.

### MD simulation

The native AF-COX-2 protein and its complexes with the hit AGP analogs (screened from the docking studies), AGP, aspirin, and rofecoxib were used to commence the MD simulation studies. GROMACS 2019.2 was used to carry out the MD simulation^63^. All the topological parameters for chemical compounds were obtained from the PRODRG server^64^. GROMOS96 54a7 force field was used to perform all the simulation studies^65^. Further, an explicit simple point charge (SPC) water model was used to solvate the system in a dodecahedron box type. The system was neutralized by the addition of counter-ions to sustain the protonated residual state at physiological pH 7.4. Subsequently, the initial steric hindrance was eliminated by the steepest descent energy method for 50,000 steps to minimize the system. Thereafter, NVT/NPT ensembles with a leap-frog integrator at 300 K temperature, and 1.0 bar pressure were used to equilibrate the system. The MD simulation was run for 100 ns for each equilibrated system with 5000 frames. The statistical MD simulation analysis was carried out through WebGRO (https://simlab.uams.edu/) by calculating the RMSD (root-means-square-deviation), RMSF (root-mean-square-fluctuation), the average area per residue, SAS (solvent–Accessible-surface) area, volume, and density, H-bonds (number of hydrogen-bonds in protein as well as between protein and ligand), and Rg (radius of gyration) value.

## Electrostatic potential energy simulation of peptide-ligand adduct

To assess the stability of adducts of AGP, hit analogs, aspirin, and rofecoxib with the selected peptide length of AF-COX-2, the EPE docking simulation study was carried out using Spartan 20. For the same, amino acid sequence length from 511 to 520 of AF-COX-2 (i.e. Val511, Gly512, Ala513, Pro514, Phe515, Ser516, Leu517, Lys518, Gly519, and Leu520) was selected as a peptide for forming the adducts with the compounds. For the same, first a model consisting of 10 amino acid residue peptide and one ligand was built. This was followed by energy minimization with the molecular mechanics option followed by the B3LYP/6-31G* single-point calculations. The energy of adduct was obtained directly from the output of the computations. Further, to calculate the electronic parameters, the data related to B3LYP/6-31G* basis set was taken as the number of unpaired electrons (multiplicity) = 0, total charge = neutral coupling, and coupling constant = empirical and stabilization energy was determined by deducing the sum of the energy of the individual constituents from the adduct energy. Then, EPE was calculated for each adduct and peptide which characterized the electron distribution on the surface of compounds^66^. Finally, the binding energy of complex compounds with peptide was calculated using the following Eq. (7).

7 $${\text{BE}}\left( {{\text{adduct}}} \right) = {\text{E}}\left( {{\text{adduct}}} \right) - \left[ {{\text{E}}\left( {{\text{compound}}} \right) + {\text{E}}\left( {{\text{peptide}}} \right)} \right]$$

### MM/GBSA rescoring study

The binding free energies in terms of molecular mechanics generalized born surface area (MM/GBSA) of AGP’s hit analogs, AGP, aspirin, and rofecoxib complexes were calculated by Fast Amber Rescoring (FAR)^67^. The ff14SB force field was used for the small molecules, whereas the Generalized Amber Force Field2 (GAFF2) was employed for protein analysis^68^. Further, the AM1-BCC method was used to assign the partial charge in the analogs through an antechamber module of Amber^69,70^.

## Results and discussion

### Selection, validation, and binding site prediction of protein

The full sequenced COX-2 protein structure AF-P35354-F1, comprising 1-604 amino acids, was used in this study, which is modeled by AlphaFold and uses a state-of-the–Art machine learning method for protein modeling. Before performing any computational analysis, this protein structure was validated by comparing the overall quality of the protein with the existing four crystallized COX-2 protein structures (PDB ID: 5F19, 5KIR, 5F1A, and 5IKQ) and a modeled structure (PDB ID: 1V0X). Supplementary Table S1 summarizes the results obtained from SAVES, ProSA, and ProQ for all the protein structures, which use ERRAT, VERIFY, PROVE, Whatcheck, Procheck, Z score, and LGscore to assess the quality of the protein by calculating the overall quality factor, compatibility of an atomic 3D model with its amino acid sequence, volumes of atoms, stereochemical parameters, and stereochemical quality. The AF-COX-2 protein structure successfully passed the VERIFY and PROVE with 97.74% overall quality factor with 90.9% residues in the most favored reason. In addition, z-score and LGscore values for the AF-COX-2 protein were found to be -8.97, and 7.639, respectively which indicates that AF-COX-2 model protein structure is of good quality^40,42^. Further, the overall quality of the AF-COX-2 protein structure was found to be the best among all other structures in terms of quality, compatibility, and stereochemistry parameters, and hence this 3D protein structure was used for further studies.

CASTp 3.0 predicted 96 possible binding pockets for the AF-COX-2 protein (Supplementary Table S2). Then, according to the shape, internal cavities, area, and volume values for the binding pockets, the amino acids from the second binding pocket (His75, Arg106, Phe191, Val214, Val330, Ile331, Tyr334, Val335, Leu338, Tyr341, Leu345, Lys346, Gln358, Asn361, Tyr371, Trp373, Lys454, Arg455, Met457, Arg499, Phe504, Glu510, Phe515, Ser516, Leu517, Leu520, Met521) were selected for further consideration. Next, binding site residues for the study were selected based on the interaction of rofecoxib and aspirin present in 5KIR and 5F19 PDB structure, respectively. Further, in the 5KIR PDB structure the binding site residues for rofecoxib were found to be Val344, Trp387, Phe518, Tyr385, 355, Ser530, Leu531, Arg120, 513, Glu524, and His90. Whereas, in the 5F19 PDB structure the aspirin binding site residues recognized as Tyr385, Ser530, Leu531, Glu524, Arg120, 513, and His 90. Therefore, to find out the more efficient compound than rofecoxib and aspirin, we have considered the binding site residues of both the compounds. Hence, we have selected the common binding residues (Tyr385, Ser530, Leu531, Arg120, and Glu524) along with the unique binding residues present on the rofecoxib and aspirn. Accordingly, we have considered His75, Arg106, Val330, Tyr334, Val335, Tyr341, Tyr371, Trp373, Arg499, Phe504, Glu510, Ser516, and Leu517 residues as binding site in this study for AF-COX-2 protein. Interestingly, CASTp 3.0 analysis revealed that all the selected binding site residues are also present in the predicted binding pocket for the AF-COX-2 and also may be important for ligand binding and protein inhibition^71^.

### Multiple sequence alignment to predict active binding site

The AF-COX-2 protein has a modeled 3D structure with no co-crystallized ligand, therefore, binding site residue prediction in the refined protein structure was carried out by comparing it with the active site of the existing crystallized PDB structure of COX-2 present in the PDB database. For the same, 5F19 and 5KIR PDB structures of COX-2 were selected which have aspirin and rofecoxib as co-crystal ligands, respectively. Based on the binding sites of both the co-crystal ligands in the protein structure, His75, Arg106, Val330, Tyr334, Tyr341, Tyr371, Trp373, Arg499, Phe504, Glu510, Ser516, and Leu517 amino acids were selected as the active sites for binding interaction studies. To verify these residues as the prominent active site, multiple sequence alignment was performed using the COX-2 protein of 10 different mammalian species, which proved that except for Arg499, all the selected amino acids are fully conserved (Supplementary Fig. S1 and Table S3). This also signifies the importance of these amino acids in various biological functions and as a potential target for drug development^47^.

### Site-directed virtual screening and molecular docking analysis

A set of 237 andrographolide analogs that consists of the common α,β-unsaturated γ-lactone moiety which is important for biological activity were screened against the full sequence validated AF-COX-2 protein to find out a potent plant based anti-inflammatory compound^25,26^. The site directed virtual screening was utilized to discover the hit AGP analogs by computational approach. It calculates the binding energy for all the subjected analogs by the results of protein–ligand interaction. Subsequently, the energetically more favorable analogs’ complexes were selected for further analysis. The results from the virtual screening analysis revealed that the lowest binding energy was found to be − 8.80 kcal/mol for PubChem ID: 132210370, while the highest binding energy was determined to be − 5.90 kcal/mol for PubChem ID:59876522 (Supplementary Table S4). Thereafter, based on the binding energy cutoff value (− 8.00 kcal/mol), 22 AGP analogs out of 237 were screened for further analysis. Therefore, these 22 AGP analogs were subjected to molecular docking studies to find out the potent COX-2 inhibitor along with AGP, and control drugs (aspirin and rofecoxib).

The binding energy values obtained from the molecular docking analysis were found to be in the range of − 9.35 kcal/mol (PubChem ID: 132210508) to − 3.2 kcal/mol (PubChem ID: 132217538) (Supplementary Table S5). In contrast, aspirin, rofecoxib, and AGP showed binding energy values of − 5.61 kcal/mol, − 8.17 kcal/mol, and − 7.95 kcal/mol, respectively. Out of 22 AGP analogs, 7 hit analogs, named A1–A7, were selected for further analysis based on the binding energy cutoff value of lesser than − 8.00 kcal/mol. In terms of binding energy, all seven hit analogs showed lesser value compared to aspirin and AGP, while five analogs (A1–A5) exhibited lesser value than rofecoxib (Table 1), indicating their potential towards COX-2 protein inhibition.

**Table 1:** Molecular docking result of the selected AGP analogs, AGP, rofecoxib, and aspirin.

PubChem ID	Compounds name	Chemical structure	IUPAC name	Binding energy (kcal/mol)
132210508	A1		3-[2-[(1R,4aR,5R,6S,8aR)-6-hydroxy-5,6,8a-trimethyl-2-methylidene-3,4,4a,5,7,8-hexahydro-1H-naphthalen-1-yl]ethylidene]-4-hydroxyoxolan-2-one	− 9.35
132210370	A2		3-[2-[(1R,4aR,5R,6S,8aR)-6-hydroxy-5,8a-dimethyl-2-methylidene-1,3,4,4a,5,6,7,8-octahydronaphthalen-1-yl]ethylidene]-4-hydroxyoxolan-2-one	− 8.92
132210409	A3		3-[2-[(1R,4aR,5R,6R,8aR)-6-hydroxy-5,6,8a-trimethyl-2-methylidene-3,4,4a,5,7,8-hexahydro-1H-naphthalen-1-yl]ethylidene]-4-hydroxyoxolan-2-one	− 8.56
132210478	A4		3-[2-[(1R,4aR,5R,6R,8aR)-6-hydroxy-5,8a-dimethyl-2-methylidene-1,3,4,4a,5,6,7,8-octahydronaphthalen-1-yl]ethylidene]-4-hydroxyoxolan-2-one	− 8.55
132210549	A5		(3E,4S)-3-[2-[(1R,4aR,5R,6S,8aR)-6-hydroxy-5,8a-dimethyl-2-methylidene-6-(trifluoromethyl)-3,4,4a,5,7,8-hexahydro-1H-naphthalen-1-yl]ethylidene]-4-hydroxyoxolan-2-one	− 8.31
59876521	A6		(6aR,7S,10bR)-7-[(2E)-2-(4-hydroxy-2-oxooxolan-3-ylidene)ethyl]-6a,10b-dimethyl-8-methylidene-1,4a,5,6,7,9,10,10a-octahydronaphtho[2,1-d][1,3]dioxin-3-one	− 8.11
59876529	A7		(3E)-3-[2-[(6aR,7S,10bR)-6a,10b-dimethyl-8-methylidene-1,4a,5,6,7,9,10,10a-octahydronaphtho[2,1-d][1,3]dioxin-7-yl]ethylidene]-4-hydroxyoxolan-2-one	− 8.10
5318517	AGP (Andrographolide)		(3E,4S)-3-[2-[(1R,4aS,5R,6R,8aS)-6-hydroxy-5-(hydroxymethyl)-5,8a-dimethyl-2-methylidene-3,4,4a,6,7,8-hexahydro-1H-naphthalen-1-yl]ethylidene]-4-hydroxyoxolan-2-one	− 7.95
5090	Rofecoxib		3-(4-methylsulfonylphenyl)-4-phenyl-2H-furan-5-one	− 8.17
2244	Aspirin		2–Acetyloxybenzoic acid	− 5.61

IUPAC,  International Union of Pure and Applied Chemistry.

### Comparative molecular interactions and binding pose analysis

**Figure 1:**
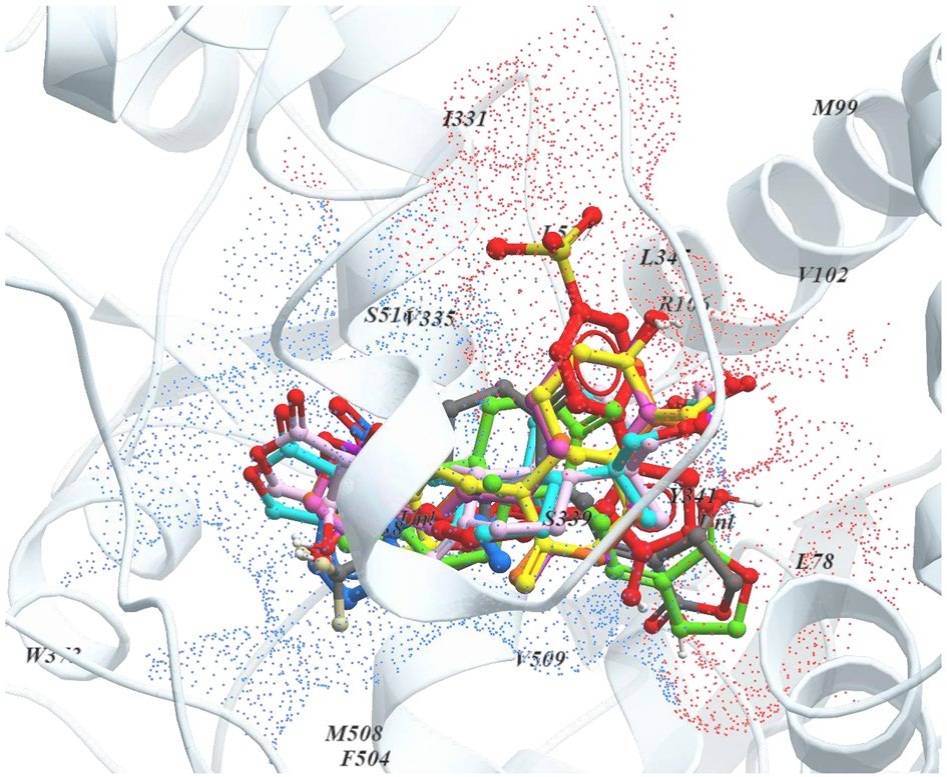
Superimposition of aspirin, rofecoxib, AGP, and hit AGP analogs at the binding site of AF-COX-2. All the chemical compounds are represented as ball and stick model, protein as ribbon representation, and interacting amino acids as single letter code. Color code: light grey = AF-COX-2 protein, blue dotted surface = aspirin binding pocket, red dotted surface = rofecoxib binding pocket, blue = aspirin, red = rofecoxib, yellow = AGP, green = A1, orange = A2, magenta = A3, hot pink = A4, dark slate grey = A5, cyan = A6, and plum = A7. This figure is plotted using ICM-Browser^52^.

**Figure 2:**
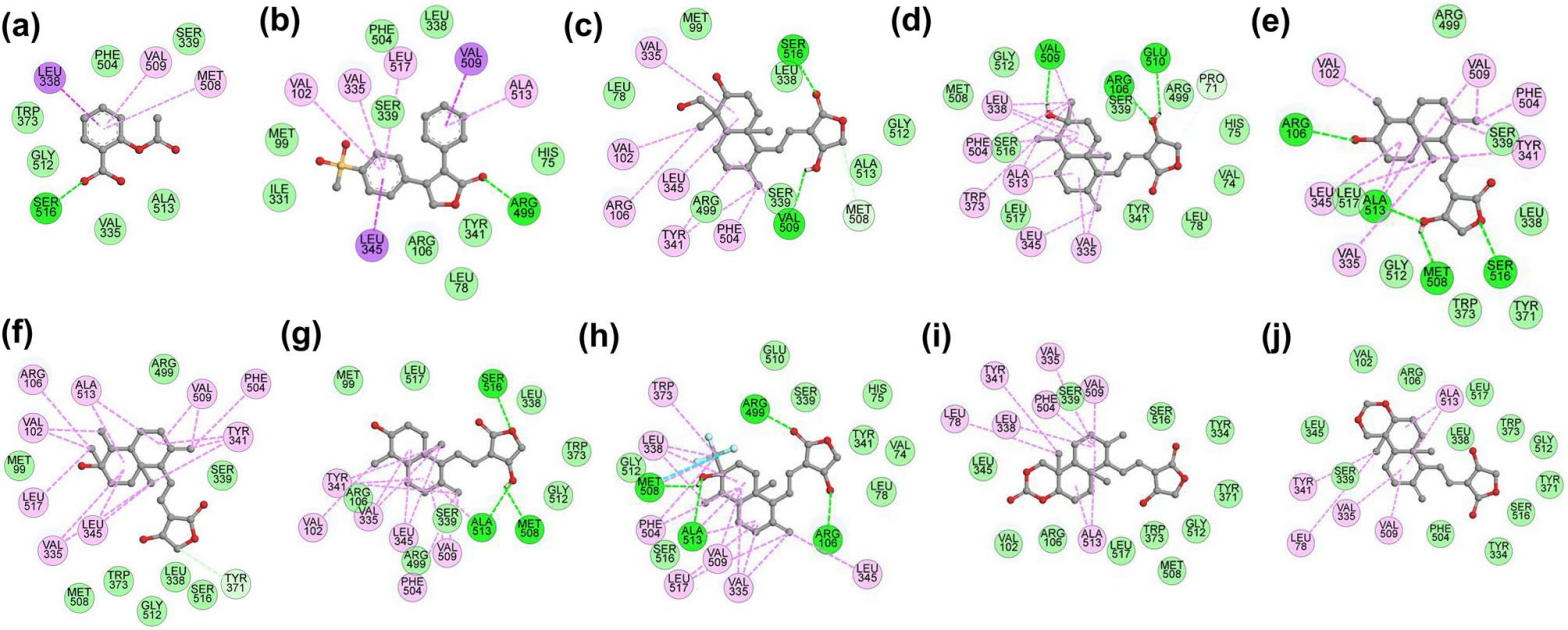
2D binding interactions of aspirin (**a**), rofecoxib (**b**), AGP (**c**), and AGP analogs (A1–A7) (**d**–**j**) with the binding site of AF-COX-2 protein. All the chemical compounds are depicted as ball and stick model; amino acids with their number are represented as a circle with three-letter code and a dashed line shows the interaction site of chemical compounds with amino acids. Color code: green = H-bonds, pink = HYD interactions.

The binding pattern of AGP and its selected seven hit analogs with respect to the existing COX-2 protein inhibitors, i.e., aspirin and rofecoxib (controls), was examined to compare the molecular binding interaction and binding pose analysis (Table 2). This analysis reveals that Val335, Leu338, Ser339, Phe504, Val509, and Ala513 amino acids are common in the interactions of all the subjected compounds with AF-COX-2 protein. In addition, Trp373, Gly512, and Ser516 amino acids are shared by aspirin, AGP, and A1–A7 analogs, whereas Leu78, Val102, Arg106, Tyr341, Leu345, Leu517 residues are occupied by rofecoxib, AGP, and A1–A7. Further, among all the interacting amino acids, Arg106, Tyr371, Arg499, Met508, Val509, Glu510, Ala513, and Ser516 residues were involved in H-bond interaction, while Leu78, Val102, Arg106, Val335, Leu338, Tyr341, Leu345, Phe504, Met508, Val509, Ala513, Leu517 residues were engaged in the HYD interaction. AGP and hit analogs A1–A5 showed 3, 3, 4, 1, 3, and 4 H-bonds, respectively, while no H-bond was formed in the case of A6 and A7 analog. Intriguingly, it is evident from the binding pattern that AGP and A1–A7 analogs share the interacting amino acid residues of both aspirin and rofecoxib binding site of AF-COX-2 protein (Figs. 1 and 2), indicating their potential as COX-2 protein inhibitor with improved binding interaction. Further, the binding orientation of AGP and hit analogs display that oxalone moiety is faced towards the aspirin binding site, while the naphthalene/naphthol moiety is positioned towards the rofecoxib binding site (Supplementary Fig. S2).

**Table 2:** Summary of comparative binding interaction analysis of aspirin, rofecoxib, AGP, and hit AGP analogs A1–7 with AF-COX-2 protein.

Compounds Name	Interacting amino acids	H-bond (distance) with interacting site	HYD bond (distance) with interacting site
Aspirin	Val335, Leu338, Ser339, Trp373, Phe504, Met508, Val509, Gly512, Ala513, Ser516	Ser516 (3.97 Å) [COOH group]	Leu338 (4.50 Å) [benzene ring], Met508 (6.33 Å) [Benzene ring], Val509 (4.59 Å) [Benzene ring]
Rofecoxib	His75, Leu78, Met99, Val102, Arg106, Ile331, Val335, Tyr341, Leu345, Leu338, Ser339, Arg499, Phe504, Val509, Ala513, Leu517	Arg499 (6.43 Å) [5-one(furan ring)]	Val102 (6.06) [Sulphonylphenyl Moiety], Val335 (6.38 Å) [Sulphonylphenyl Moiety], Leu345 (5.68 Å) [Phenyl Moiety], Val509 (4.57 Å) [Phenyl Moiety], Ala513 (5.11 Å) [Phenyl Moiety], Leu517 (6.75 Å) [Sulphonylphenyl Moiety]
AGP	Leu78, Met99, Val102, Arg106, Val335, Leu338, Ser339, Tyr341, Leu345, Arg499, Phe504, Met508, Val509, Gly512, Ala513, Ser516	Ser516 (4.05 Å) [2-one (oxolane Moiety)], Met508 (4.99 Å) [Oxolane Moiety], Val509 (2.83 Å) [4-hydroxy (oxolane Moiety)]	Val102 (4.43 Å) [5-methyl], Arg106 (4.40 Å) [5-methyl], Val335 (5.36 Å) [Naphthalene moiety], Tyr341 (4.60 Å; 5.60 Å; 6.48 Å) [Naphthalene moiety], Leu345 (5.33 Å; 6.14 Å) [Naphthalene moiety], Phe504 (5.79 Å) [2-methylidene], Val509 (4.48 Å; 5.33 Å) [Naphthalene moiety]
A1	Pro71, Val74, His75, Leu78, Arg106, Val335, Leu338, Ser339, Tyr341, Leu345, Trp373, Arg499, Phe504, Met508, Val509, Glu510, Gly512, Ala513, Ser516, Leu517	Arg106 (6.44 Å) [4-hydroxy-oxolane], Val509 (2.84 Å) [6-hydroxy], Glu510 (4.69 Å) [4-hydroxy-oxolane]	Val335 (4.53 Å; 5.47 Å) [Naphthalene moiety], Leu338 (5.24 Å) [8a-methyl], Leu345 (5.39 Å) [8a-methyl], Phe504 (6.05 Å) [Naphthalene moiety], Val509 (3.35 Å) [Naphthalene moiety], Ala513 (3.49 Å; 4.61 Å; 4.92 Å) [Naphthalene moiety]
A2	Val102, Arg106, Val335, Leu338, Ser339, Tyr341, Leu345, Tyr371, Trp373, Arg499, Phe504, Met508, Val509, Gly512, Ala513, Ser516, Leu517	Arg106 (3.68 Å) [6-hydoxy naphthalene], Met508 (3.66 Å) [4-hydroxy oxolane], Ala513 (4.06 Å) [4-hydroxy oxolane], Ser516 (4.30 Å) [Oxolane moiety]	Val102 (4.44 Å) [5-methyl], Val335 (5.10 Å; 5.46 Å) [8a-methyl; Naphthalene moiety], Tyr341 (5.39 Å; 5.85 Å; 6.99 Å) [8a-methyl; 2-methylidene; Naphthalene moiety], Leu345 (5.50 Å) [8a-methyl], Phe504 (5.79 Å) [2-methylidene], Val509 (4.32 Å; 4.94 Å) [2-methylidene; Naphthalene moiety], Ala513 (4.81 Å; 5.39 Å) [Naphthalene moiety]
A3	Met99, Val102, Arg106, Val335, Leu338, Ser339, Tyr341, Leu345, Tyr371, Trp373, Arg499, Phe504, Met508, Val509, Gly512, Ala513, Ser516, Leu517	Tyr371 (5.81 Å) [Oxolane moiety]	Val102 (4.35 Å) [5-methyl], Val335 (5.07 Å; 5.41 Å) [8a-methyl; Naphthalene moiety], Tyr341 (5.36 Å; 5.90 Å; 7.01 Å) [8a-methyl; 2-methylidene; Naphthalene moiety], Leu345 (5.64 Å) [8a-methyl], Phe504 (5.74 Å) [2-methylidene], Val509 (4.15 Å; 4.82 Å) [2-methylidene; Naphthalene moiety], Ala513 (4.80 Å; 5.37 Å) [Naphthalene moiety]
A4	Met99, Val102, Arg106, Val335, Leu338, Ser339, Tyr341, Leu345, Trp373, Arg499, Phe504, Met508, Val509, Gly512, Ala513, Ser516, Leu517	Met508 (3.73 Å) [4-hydroxy oxolane], Ala513 (4.01 Å) 4-hydroxy oxolane], Ser516 (4.45 Å) [Oxolane moiety]	Val102 (4.35 Å) [5-methyl], Val335 (5.17 Å; 5.38 Å) [8a-methyl; Naphthalene moiety], Tyr341 (5.20 Å; 5.84 Å; 6.90 Å) [8a-methyl; 2-methylidene; Naphthalene moiety], Leu345 (5.56 Å) [8a-methyl], Phe504 (5.74 Å) [2-methylidene], Val509 (4.22 Å; 4.92 Å) [2-methylidene; Naphthalene moiety], Ala513 (4.93 Å) [Naphthalene moiety]
A5	Val74, His75, Leu78, Arg106, Val335, Leu338, Ser339, Tyr341, Leu345, Trp373, Arg499, Phe504, Met508, Val509, Glu510, Gly512, Ala513, Ser516, Leu517	Arg106 (5.76 Å) [6-hydoxy naphthalene], Arg499 (6.55 Å) [5-one(furan ring)], Met508 (3.91 Å) [6-hydroxy naphthalene], Ala513 (3.86 Å) [6-hydroxy naphthalene]	Val335 (4.32 Å; 5.20 Å; 5.24 Å; 5.85 Å) [2-methylidene; Naphthalene moiety], Leu338 (4.81 Å; 5.86 Å) [5 and 8a-methyl], Leu345 (5.60 Å) [2-methylidene] Val509 (4.70 Å) [Naphthalene moiety], Ala513 (4.27 Å; 4.39 Å) [Naphthalene moiety], Leu517 (6.06 Å; 7.03 Å) [2-methylidene; Naphthalene moiety]
A6	Leu78, Val102, Arg106, Tyr334, Val335, Leu338, Ser339, Tyr341, Leu345, Tyr371, Trp373, Phe504, Met508, Val509, Gly512, Ala513, Ser516, Leu517	–	Leu78 (5.95 Å) [10b-methyl], Val335 (4.92 Å; 5.76 Å) [Naphthalene], Leu338 (4.52 Å; 5.72 Å) [8-methylidene; naphthalene], Tyr341 (5.75 Å, 5.99 Å) [10b-methyl; 1,3-dioxin], Leu345 (6.49 Å) [1,3-dioxin], Phe504 (5.01 Å; 7.52 Å) [8-methylidene; naphthalene], Val509 (3.68 Å; 3.86 Å; 4.93 Å) [8-methylidene; 6a-methyl; naphthalene], Ala513 (3.60 Å; 3.95 Å; 4.09 Å) [6a-methyl; naphthalene]
A7	Leu78, Val102, Arg106, Tyr334, Val335, Leu338, Ser339, Tyr341, Leu345, Tyr371, Trp373, Phe504, Val509, Gly512, Ala513, Ser516, Leu517	–	Leu78 (5.85 Å) [10b methyl], Val335 (5.80 Å) [Naphthol], Leu338 (4.39 Å) [8-methylidene], Tyr341 (5.91 Å) [10b methyl], Phe504 (5.24 Å) [8-methylidene], Val509 (3.94 Å; 4.00 Å; 5.20 Å) [8-methylidene; 6a-methyl; naphthalene], Ala513 (3.62 Å; 4.10 Å; 6.26 Å) [6a-methyl; naphthol]

H bond,  Hydrogen bond; HYD,  Hydrophobic.

### ADMET prediction

The physicochemical, absorption, distribution, metabolism, excretion, and toxicological properties of AGP and A1–A7 analogs were predicted and compared with aspirin and rofecoxib to evaluate their drug-likeness (Table 3). Physicochemical properties such as molecular weight (MW), number of H-bond acceptors (nHA), number of H-bond donors (nHD), topological polar surface area (TPSA), and the logarithm of the n-octanol/water distribution coefficient (logP) were calculated for all the selected compounds. Further, medicinal chemistry properties, i.e. natural product-likeness score (NPscore), Lipinski rule, and Pfizer rule, were analyzed for all the compounds. Except for rofecoxib and A5 analog, all the compounds fulfilled the criteria for physicochemical and medicinal chemistry properties for drug-like candidates. Furthermore, the absorption properties such as Caco-2 (the human colon adenocarcinoma cell lines) permeability, Pgp-inhibitory activity (inhibitor of P-glycoprotein), human intestinal absorption (HIA range 0–0.3), and F_30%_ (the human oral bioavailability 30%) were also computed for all the compounds. Except for rofecoxib and A6 analog, all the compounds fulfilled the criteria to be used as orally active drugs. In addition, distribution properties for example plasma protein binding (PPB), the volume of distribution (VD), blood–brain barrier (BBB) penetration, and fraction unbound in plasma (Fu) were predicted for all the studied molecules. The distribution properties of AGP and A1–A7 analogs were found to be in the acceptable range for a drug-like candidate. Moreover, the cumulative analysis of metabolism and excretion properties also favored the AGP and A1–A7 analog as potential drug candidates. Further, the toxicological properties assessment also predicted the non-toxic nature of the A1–A7 AGP analogs.

**Table 3:** Description of physicochemical, medicinal, absorption, distribution, metabolism, excretion, and toxicological properties with their permissible range of rofecoxib, aspirin, AGP, and A1–A7 AGP analogs.

Category	Parameters	Permissible range	Rofecoxib	Aspirin	AGP	A1	A2	A3	A4	A5	A6	A7
Physicochemical property	MW	100 ~ 600	314.06	180.04	350.21	334.21	320.2	334.21	320.2	388.19	376.19	362.21
	HA	< 38	22	13	25	24	23	24	23	27	27	26
	nHA	0–12	4	4	5	4	4	4	4	4	6	5
	nHD	0–7	1	1	3	2	2	2	2	2	1	1
	TPSA	0–140	67.51	63.6	86.99	66.76	66.76	66.76	66.76	66.76	82.06	64.99
	logP (log mol/L)	0–3	3.014	1.237	1.58	2.763	2.435	2.763	2.435	3.229	2.458	2.556
Medicinal chemistry	NPscore	− 5 to 5	− 0.256	0.122	3.02	2.793	2.936	2.793	2.936	2.224	2.591	2.741
	Lipinski Rule	MW ≤ 500, logP ≤ 5, nHA ≤ 10, nHD ≤ 5, < 2 violations	Accepted	Accepted	Accepted	Accepted	Accepted	Accepted	Accepted	Accepted	Accepted	Accepted
	Pfizer Rule	logP > 3, TPSA < 75	Rejected	Accepted	Accepted	Accepted	Accepted	Accepted	Accepted	Rejected	Accepted	Accepted
Absorption	Caco-2 Permeability (log cm/s)	> − 5.15	− 5.598	− 5.062	− 4.793	− 4.604	− 4.647	− 4.604	− 4.647	− 4.665	− 4.732	− 4.822
	Pgp-inhibitor	0–0.3	0.01	0	0	0	0	0	0	0	0.001	0.002
	HIA	0–0.3	0.005	0.007	0.245	0.028	0.217	0.028	0.217	0.01	0.007	0.008
	F_30%_	0–0.3	0.003	0.003	0.036	0.006	0.049	0.006	0.049	0.007	0.501	0.02
Distribution	PPB	≤ 0.90	0.99	0.59	0.30	0.57	0.52	0.57	0.52	0.73	0.43	0.47
	VD (L/kg)	0.04–20	0.626	0.234	0.612	0.811	0.885	0.811	0.885	0.803	0.839	0.997
	BBB Penetration (cm/s)	0 to 1	0.037	0.661	0.955	0.996	0.987	0.996	0.987	0.987	0.995	0.983
	Fu	≥ 5%	0.02	0.61	0.67	0.52	0.54	0.52	0.54	0.31	0.63	0.55
Metabolism	CYP inhibitor	0–1	0.146	0.016	0.167	0.407	0.155	0.407	0.155	0.351	0.466	0.412
	CYP substrate	0–1	0.358	0.114	0.219	0.283	0.252	0.283	0.252	0.32	0.26	0.246
Excretion	CL (ml/min/kg)	> 15 high clearance; 5–15 moderate clearance; < 5 low clearance	0.535	2.736	8.907	14.47	14.712	14.47	14.712	9.945	13.704	13.431
	T 1/2	0–0.3 excellent 0.3–0.7 medium; 0.7–1.0 poor	0.116	0.91	0.249	0.119	0.134	0.119	0.134	0.04	0.121	0.087
Toxicology	hERG Blockers	0–0.3 excellent 0.3–0.7 medium; 0.7–1.0 poor	0.087	0.015	0.003	0.004	0.004	0.004	0.004	0.005	0.001	0.002
	H-HT	0–0.3 excellent 0.3–0.7 medium; 0.7–1.0 poor	0.592	0.258	0.012	0.017	0.017	0.017	0.017	0.029	0.012	0.015
	AMES Toxicity	0–0.3 excellent 0.3–0.7 medium; 0.7–1.0 poor	0.006	0.013	0.327	0.266	0.227	0.266	0.227	0.158	0.065	0.271
	Skin Sensitization	0–0.3 excellent 0.3–0.7 medium; 0.7–1.0 poor	0.048	0.511	0.026	0.018	0.022	0.018	0.022	0.018	0.032	0.023
	IGC_50_ {− log10[(mg/L)/(1000*MW)]}	–	3.199	2.661	4.909	4.825	4.967	4.825	4.967	5.002	5.28	5.257
	LC_50_ {− log10[(mg/L)/(1000*MW)]}	–	4.275	3.748	6.605	6.114	6.293	6.114	6.293	6.461	6.613	6.973

MW, Molecular Weight; nHA, Number of hydrogen bond acceptors; nHD, Number of hydrogen bond donors; TPSA, Topological polar surface area; logP, The logarithm of the n-octanol/water distribution coefficient; NPscore, Natural Product-likeness score; Caco-2 Permeability, The human colon adenocarcinoma cell lines; Pgp-inhibitor, Inhibitor of P-glycoprotein; HIA, Human intestinal absorption; F 30%, The human oral bioavailability 30%; PPB, Plasma protein binding; VD, Volume Distribution; BBB, blood–brain barrier; Fu, fraction unbound in plasma; CYP, cytochrome; CL, clearance; hERG, human ether–A-go-go related gene; H-HT, human hepatotoxicity; IGC_50_, 50% inhibition growth concentration; LC_50_, 50% lethal concentration.

### Ligand efficiency metrics

The ligand binding quality of the selected was calculated based on the molecular mass and lipophilicity with the context of the particular protein target. Typically, a low Ki value refers to high potency and it should be in the micromolar range for a molecule to be qualified as a hit or lead compound^55^. Ligand efficiency (threshold value = 0.3 kcal/mol/HA) gives an idea about how well a compound binds for its size with the protein^56^. Ligand lipophilic efficiency (threshold value = 3) is used to measure the affinity of a molecule towards lipophilicity which is the major factor for the promiscuity of the selective LLE and optimized compounds^54,56,57^. LE_Scale is a size-independent scaling function of ligand efficiency and is derived by fitting an exponential function to the maximal ligand efficiency values observed for a given heavy atom (HA). Fit quality (threshold value = 0.8), also called as scaled ligand efficiency, includes ligand efficiency and size into a single metric and results by dividing the observed LE with LE_scale^55,56,58^. Lastly, LELP (acceptable range = -10 to + 10) was calculated which presents a metric that indicates the price of ligand efficiency paid in lipophilicity^54,55^. Further, binding energy (BE) obtained from the docking studies (Table 1), HA and LogP acquired from physicochemical properties prediction (Table 3) were taken for the calculation of all the LE metrics. It can be observed from Table 4 that except for A5 and controls (aspirin and rofecoxib), all the screened analogs show the respective ligand efficiency metrics values which surpass the minimum thresholds and to be called as HIT analogs. Also, in terms of inhibition constant, analogs A1–A5 displayed a Ki value smaller than the controls (aspirin Ki = 75.604 µM and rofecoxib Ki = 0.995 µM) and AGP (Ki = 1.440 µM) indicating their greater binding affinity towards the inhibition of COX-2 protein than controls and AGP.

**Table 4:** Ligand efficiency metrics of the screened andrographolide analogs and controls (aspirin and rofecoxib).

Compounds Name	Inhibition constant (Ki) (µM)*	Ligand Efficiency (LE*) kcal/mol/HA	Ligand Lipophilic Efficiency (LLE)*	LE_Scale*	Fit Quality (FQ)*	Ligand Efficiency Lipophilic Price (LELP)*
A1	0.135	0.389	4.107	0.404	0.963	7.102
A2	0.280	0.388	4.118	0.416	0.933	6.276
A3	0.515	0.357	3.525	0.404	0.884	7.739
A4	0.525	0.372	3.844	0.416	0.894	6.546
A5	0.783	0.308	2.877	0.369	0.835	10.484
A6	1.100	0.300	3.500	0.369	0.813	8.193
A7	1.122	0.312	3.393	0.380	0.821	8.192
AGP	1.440	0.318	4.261	0.392	0.811	4.969
Rofecoxib	0.995	0.371	2.987	0.429	0.865	8.124
Aspirin	75.604	0.432	2.883	0.559	0.773	2.863

*Ki = exp(Binding Energy/RT); LE = -BE / HA; HA = Heavy atoms; LLE = -logKi – LogP;

LE_SCALE = 0.873e^(− 0.026 × HA) – 0.064; FQ = LE ÷ LE_SCALE; LELP = LogP ÷ LE.

### Interpretation of quantum mechanical descriptors

**Figure 3:**
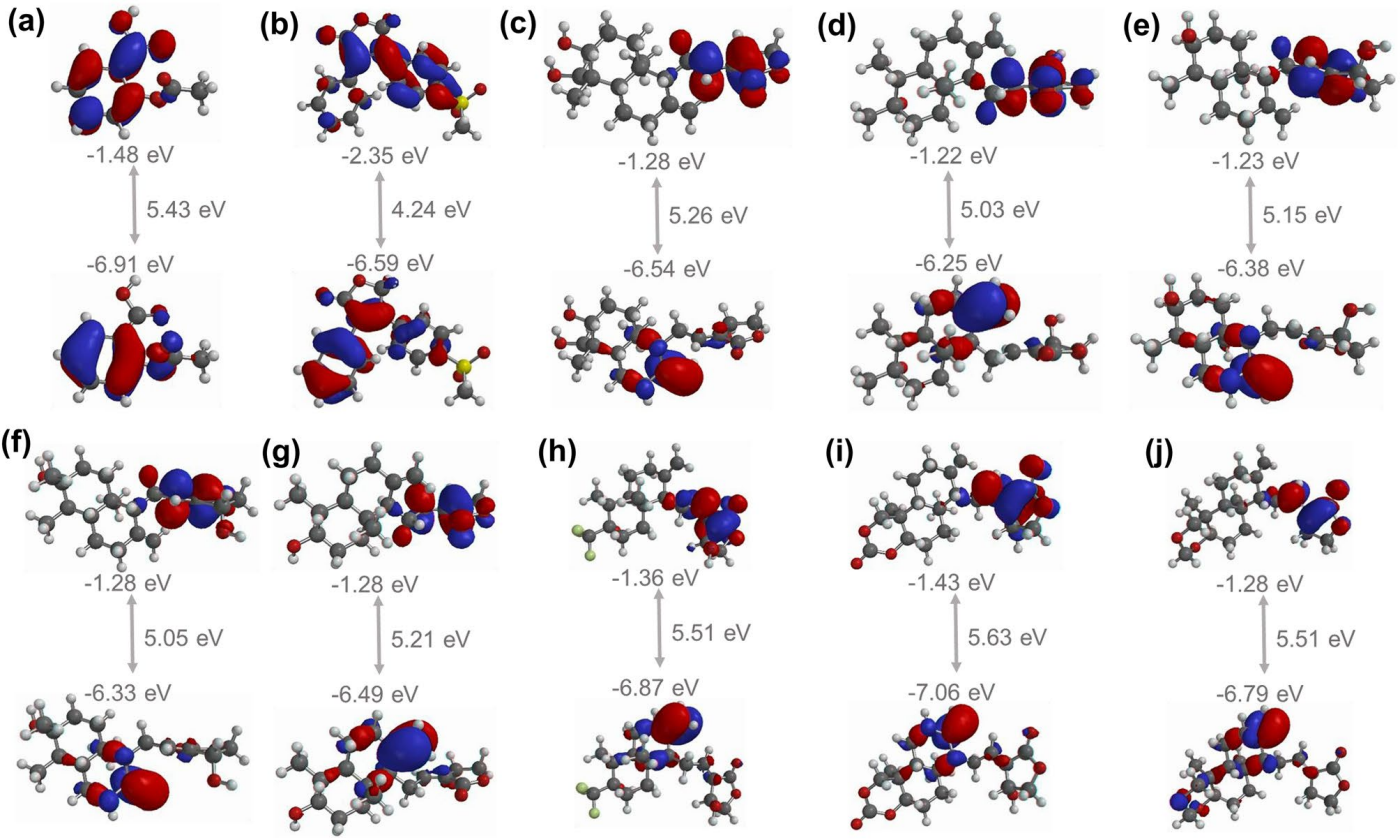
Illustration of HOMO and LUMO molecular orbitals of aspirin (**a**), rofecoxib (**b**), AGP (**c**), and AGP’s hit analogs {A1–A7} (**d**–**j**) with their HLG (HOMO–LUMO-GAP). The upper structure shows LUMO while the lower structure depicted HOMO orbitals. Color code: red = most negative potential; blue = most positive potential.

The frontier molecular orbitals (FMOs) such as HOMO, LUMO, and HLG were calculated to anticipate the electronic properties of the AGP, hit analogs, aspirin, and rofecoxib against AF-COX-2. In the case of AGP and A1–A7 analogs, HOMO and LUMO orbitals locate on the naphthalene/naphthol and oxalone moiety, respectively, whereas, in the case of aspirin, HOMO and LUMO orbitals localize on the benzoic acid part of the structure. In contrast, HOMO orbitals confine to the phenyl-furan moiety while the LUMO orbitals position on the methylsulfonylpheny-furan moiety of rofecoxib (Fig. 3). The HLG between two energetic states, i.e. HOMO and LUMO, explains the chemical reactivity of molecules where the lowest HLG value infers greater chemical reactivity and biological activity, and lesser stability of the compound by making the flow of electrons easier^72^. It also plays an important role in protein–ligand complex stabilization^73^. The HLG values were found to be in the range of 4.24 to 5.63 eV for all the investigated compounds with the order of A6 (5.63 eV) > A7 (5.51 eV) = A5 (5.51 eV) > aspirin (5.43 eV) > AGP (5.26 eV) > A4 (5.21 eV) > A2 (5.15 eV) > A3 (5.05 eV) > A1 (5.03 eV) > rofecoxib (4.24 eV). Based on the HLG value, both A1 and A3 show the most chemical reactivity, whereas A6 is the least reactive and most stable.

### Electrostatic potential energy map of the chemical compounds

**Figure 4:**
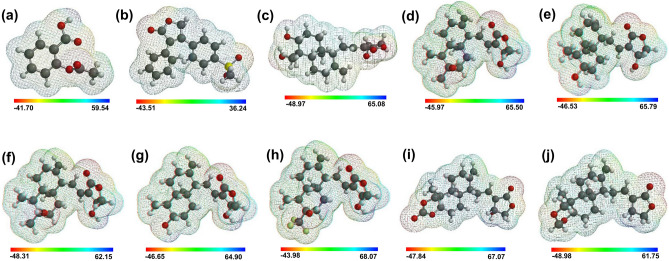
Electrostatic potential energy maps of aspirin (**a**), rofecoxib (**b**), AGP (**c**), and hit AGP analogs {A1–A7} (**d**–**j**). The color code for EPE contours maps shows the most negative value in red to the most positive value in blue color; The order of potential is: blue (most + ve) > green > yellow > orange > red (most -ve).

The EPE map is used to predict the active sites for intermolecular interactions as a spatial electron density distribution over the chemical compounds^74,75^. Figure 4 shows the EPE map of aspirin, rofecoxib, AGP, and A1–A7 in which the blue color region represents the most positive potential that is responsible for nucleophilic interaction while the red color indicates the most negative potential that is the site for electrophilic interaction. It is evident from the EPE map analysis that acetyloxy and carboxyl groups in aspirin, methylsulfonyl and furan groups in rofecoxib, and oxalone and hydroxyl groups of naphthalene moiety in AGP and hit analogs are in the negative potential (red region) and thus these are the prominent site for the electrophilic interactions. In contrast, the positive potential (blue region), which is a potential site for nucleophilic interactions, lies around the hydrogen of the carboxyl group in aspirin, phenyl group of rofecoxib, and naphthalene or naphthol moiety in AGP and A1–A7 analogs. The positive EPE (kcal/mol) were found to be in the order of rofecoxib (36.24) < aspirin (59.54) < A7 (61.75) < A3 (62.15) < A4 (64.90) < AGP (65.08) < A1 (65.50) < A2 (65.79) < A6 (67.07) < A5 (68.07). The negative EPE (kcal/mol) trend was different and followed the order of A7 (− 48.98) > AGP (48.97) > A3 (48.31) > A6 (-47.84) > A4 (− 46.65) > A2 (− 46.53) > A1(− 45.97) > A5 (− 43.98) > rofecoxib (−43.51) > aspirin (− 41.70) (Fig. 4). As the negative value of EPE favors the stronger H-bond formation^76,77^, the AGP and its A1–A7 hit analogs showed better potential towards stronger H-bond interaction compared to the aspirin and rofecoxib.

### Structure activity relationship (SAR) of the screened AGP analogs

Structurally, all the screened AGP analogs share the main chemical scaffold which consists of two cyclic groups (i.e. naphthalene/naphthol and oxalone) linked with ethyledine moiety. Bicyclic groups containing compounds manifest a myriad of biological activities^78^. These bicyclic groups containing drugs predominantly interact with proteins through hydrophobic, and aromatic residues. Further, it has also been demonstrated that the bicyclic compounds interact with the polar groups such as hydroxyl, and amides of the amino acid^79^. In this study, amongst the screened AGP analogs, there are two stereoisomers A1 (6S)/A3 (6R), and A2 (6S)/A4 (6R), whereas, analogs A5, A6, and A7 are distinct from each other. Compared to AGP, analogs A1 to A5 structurally differ at 5 and 6 positions of naphthalene moiety, while the rest of the chemical scaffold is the same. In place of naphthalene, analogs A6 and A7 possessed naphthol moiety and has a different group at the 3rd position of the naphthol ring. Further, molecular interaction analysis revealed that amongst screened analogs naphthalene moiety-containing compounds showed more affinity compared to naphthol towards the COX-2 protein in terms of binding energy, H-bonds and HYD interactions (Table 1 and 2). It is evident from Fig. 2 and Table 2 of the molecular interaction analysis that the oxolane moiety is mainly involved in the H-bond interaction while naphthalene/naphthol moiety mainly concerned with the HYD interactions. This was further confirmed by quantum mechanical descriptor analysis. Structurally, compared to AGP, analog A1–A4 has one less –OH group at the 5^th^ position in the naphthalene moiety which increases the duration of action of these analogs which has been proved by ADMET prediction (Table 3). This further increases the HYD interaction of these types of analogs with the COX-2 protein and also increases the affinity with the receptor^80^. Therefore, based on the above relationship between the chemical structure features and the results obtained from the molecular docking and DFT analysis, we concluded that screened AGP analogs shared improved anti-inflammatory activity compared to AGP and the reference drugs (aspirin and rofecoxib).

### MD Simulation

MD simulation analysis was carried out for AGP and hit analogs’ complexes with AF-COX-2 protein along with appropriate controls {AF-COX-2 protein alone (neutral control) and its complex with aspirin and rofecoxib (positive control)}. The MD simulation trajectory analysis of AGP and hit analogs’ complex was compared with native AF-COX-2 protein, aspirin, and rofecoxib complex in terms of RMSD, RMSF, residual area, Rg, SAS area, SAS volume, SAS density, protein H-bonds, and protein–ligand complex H-bonds.

**Figure 5:**
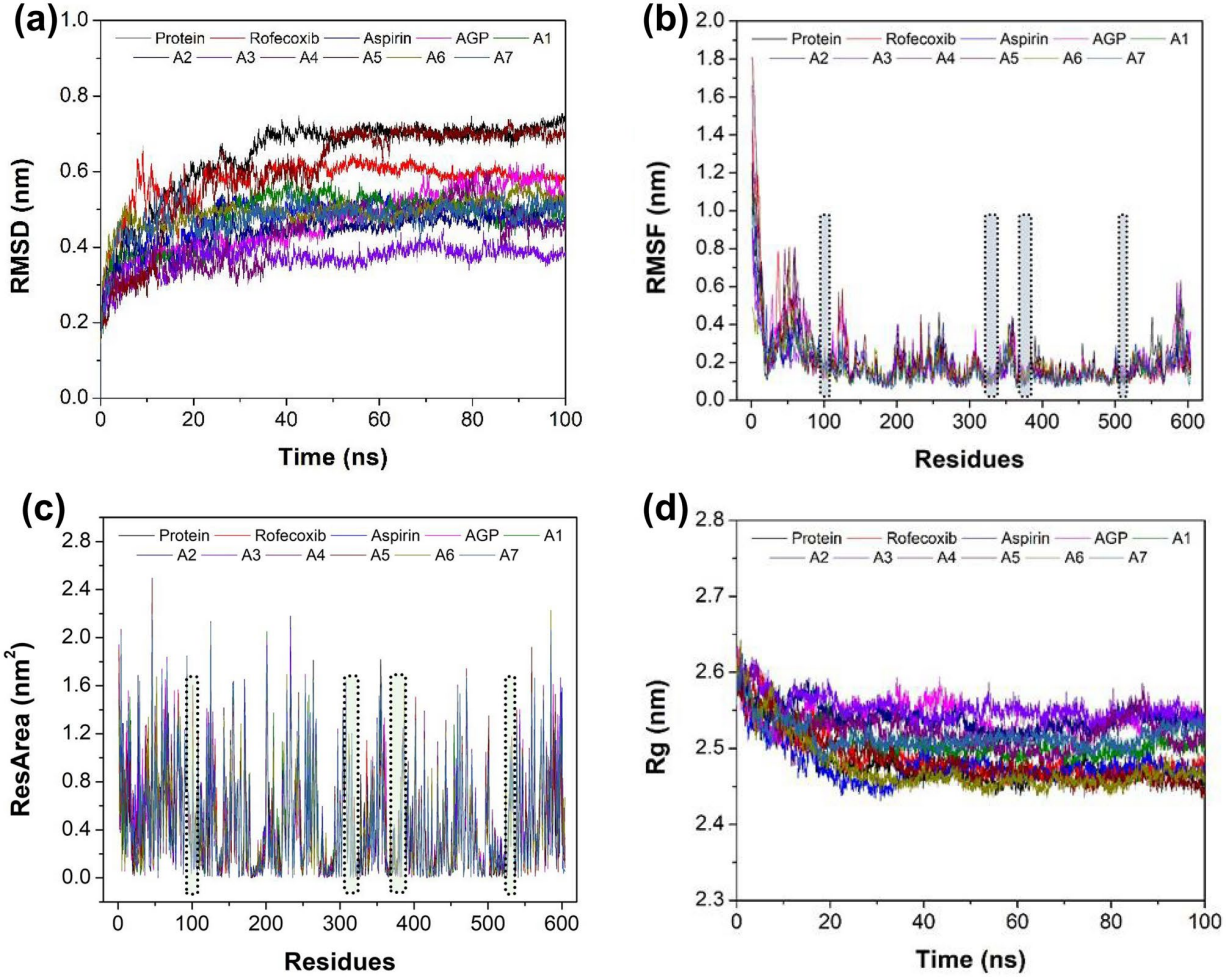
Pictorial representation of MD simulation analysis results in terms of RMSD (**a**), RMSF (**b**), SAS area for each residue (**c**), and Rg (**d**) of the native AF-COX-2 protein and its complex with subjected ligands.

The RMSD measures the protein conformation by calculating the average distance between the backbone atoms of a protein during the simulation and assessing the structural stability. The average RMSD values for the AGP and hit analogs were found to be lower than the neutral control (native protein). In comparison with positive controls, the average RMSD value was observed to be lesser in analog A2, A3, and A4. The least value RMSD value was observed for the analog A3 indicating its ability to form a stable adduct with AF-COX-2 protein (Fig. 5a and Supplementary Table S6). The RMSF is calculated for the residual mobility of each protein–ligand complex as well as the native protein. No noticeable difference was found between the residual fluctuation profiles of all the complexes (Fig. 5b). This may be ascribed to the formation of H-bonds and van der Waal interactions between amino acid residues and ligands. As shown in Fig. 5b, the active binding site regions have lower fluctuations indicating their stability in the AF-COX-2 protein. Further, the average RMSF was observed to be lower in the A1, A3, A6, and A7 complexes than in the neutral control as well as the positive control (Supplementary Table S6). The individual RMSF for the interacting amino acid residues in each simulation complex system was computed and compared with the respective average RMSF value for the simulation system. Further, from the Supplementary Table S6, the average RMSF range for all the studied simulation systems was found to be 0.16 ± 0.10 to 0.21 ± 0.18 nm. Also, it is evident that except Arg106, Ser339, and Phe 504, rest of the interacting amino acid exhibited the RMSF value lower than 0.21 nm (which is highest average RMSF value amongst all the simulation system) (Supplementary Table S7). This means that the interacting binding residues are quite stable during the MD simulation. Moreover, the lower average RMSF value for the analogs A1, A2, A3, A6 and A7 than the protein demonstrate that the analogs are capable of forming stable interactions with the protein during MD simulation. This RMSF study suggested the ability of these four AGP analogs towards the formation of stable interaction with AF-COX-2 protein during the period of simulation.

The SAS area for each residue during the simulation was also computed in order to calculate the area of each residue that is accessible for solvent (Fig. 5c)^81^. The SAS value per residue was found to be in the range of 0–2.49 nm^2^. It is evident that a few residues of the AF-COX-2 protein are not accessible for solvents such as Asn181 in rofecoxib and A2 complex, and Lys237 in A7 complex. Further, amongst all the studied analogs and control compounds, the average value of SAS area per residue was found to be highest for A3. Further, the maximum value of SAS area per residue (nm^2^) was found to be in the order of A4 (2.49) > A5 (2.44) > A3 (2.27) > A6 (2.22) > Rofecoxib (2.21) > A7 (2.19) > A2 (2.18) > A1 (2.11) > AGP (2.06) > aspirin (2.06) > protein (2.05) which signifies that analogs A3–A6 have the better accessibility for the solvent than the other complexes as well as native protein (Supplementary Table S8).

The Rg analysis was carried out to calculate the level of compactness in terms of protein folding and unfolding of the AF-COX-2 protein structure in the presence and absence of the ligands. The average Rg value for analog A3 and A6 was found to be equivalent to the Rg value of native protein (neutral control) but lesser than the positive control. This suggests a more stable and compact protein–ligand complex formation compared to other analogs and positive controls (Fig. 5d and Supplementary Table S8)^82^.

**Figure 6:**
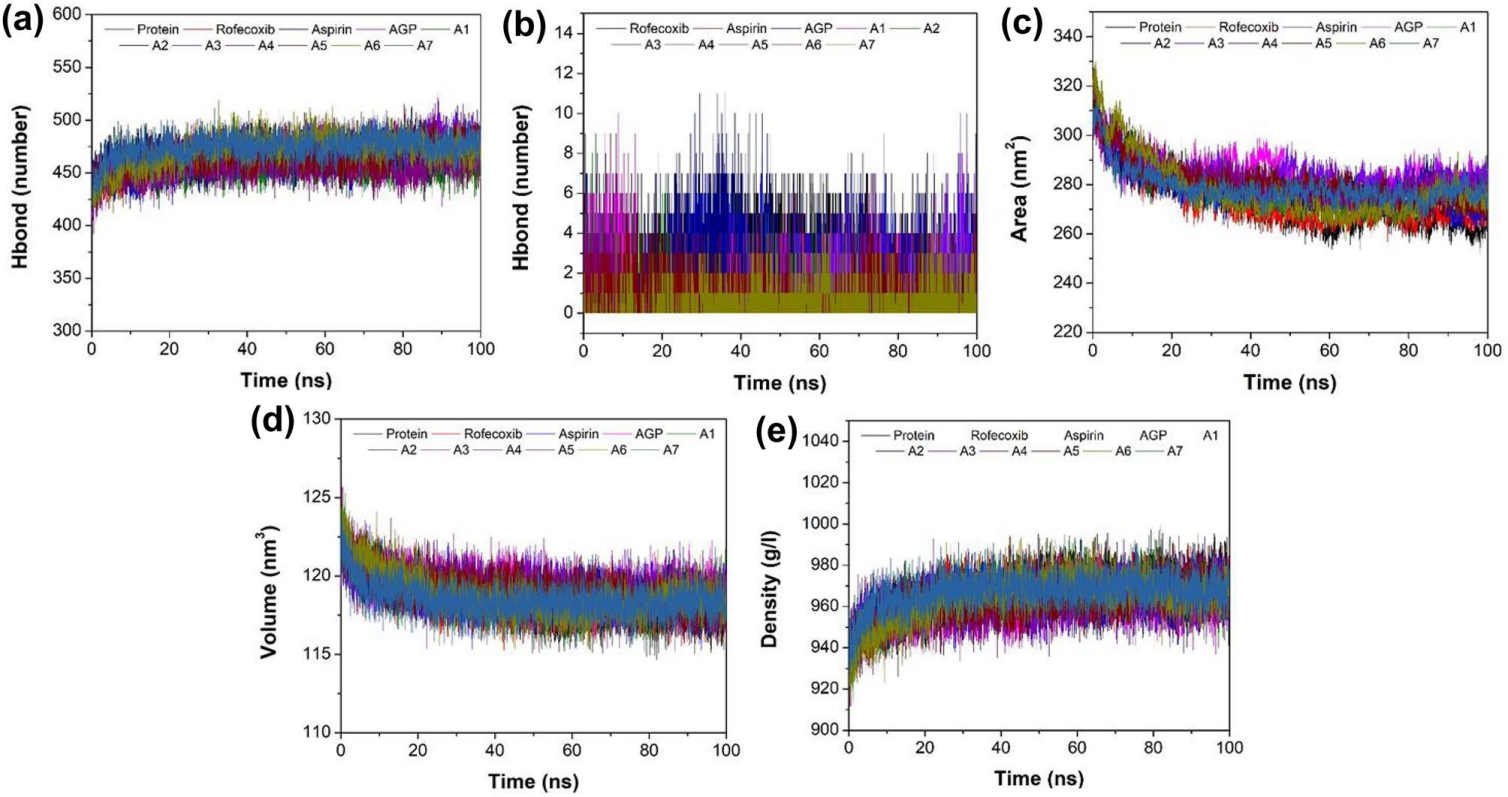
Graphical illustration of MD simulation studies of native COX-2 protein, and its complexes with aspirin, rofecoxib, AGP, and hit AGP analogs. (**a**) Number of H-bonds in protein, (**b**) number of H-bonds between protein and ligand, (**c**) SAS area, (**d**) SAS volume, and (**e**) SAS density.

The Number of H-bonds in protein as well as between protein and ligand play a significant role in the stability of complexes, therefore, H-bond analysis was performed for native AF-COX-2 and its complexes with AGP, hit analogs, aspirin, and rofecoxib. It is evident from Fig. 6a that the analog A3 complex exhibited a maximum number of H bonds in protein, whereas, AGP and A3 complexes exhibited the maximum number of protein–ligand H-bonds as compared to other analogs and positive (Fig. 6b). This suggests the stronger and more efficient binding of the analog A3 with AF-COX-2 protein which is in good agreement with the results obtained from RMSD and RMSF analysis (Supplementary Table S6).

SASA, the area of a complex surface that is accessible to a water solvent, is used to predict the level of conformational variations that occur through the interaction between proteins and ligands. Figure 6c shows the SASA plot for all the hit analogs, AGP, neutral, and positive control. The average SASA value for all the hit AGP analogs along with AGP was found to be more than the neutral control (native protein SASA = 273.57 nm^2^). Whereas, as compared to the positive control (rofecoxib and aspirin complex SASA = 273.13 and 279.12 nm^2^, respectively), AGP, A2, A3, A4, and A5 showed greater SASA values (i.e. > 279.12 nm^2^) (Supplementary Table S9). Amongst all the hit analogs and AGP, A3 exhibited the highest SASA value = 284.59 nm^2^. Conclusively, the A3 analog forms a relatively stable complex with AF-COX-2 protein after the interaction. Further, solvent–Accessible surface volume (V_sas_), the volume enclosed by the center of a solvent probe rolling around the protein, was also calculated for all the studied ligands which signifies the stability of the complex system^83^. Typically, it measures the effect of forces on the protein surfaces exerted by solvents followed by the protein-solvent interactions. Also, V_sas_ is an alternative to refine the SASA term, in order to include the influence of the solvents’ effect on the protein’s interior and also define the interaction between the protein and solvent^84^. It is an accurate and fast application to examine geometric volumes of the proteins. In addition, it can be used to calculate the volume that changes due to the interaction of protein–ligand complex with solvent^85^. V_sas_ along with SASA provides a better acquiescence of implicit and explicit nonpolar solvent forces on the protein and its effect on the protein folding^86^. As shown in Fig. 6d and Supplementary Table S9, AGP, A3, A4, and A5 analog complex showed a greater value of V_sas_ compared to all controls, while the highest value was observed for the A3 analog complex (119.24 nm\s3\n). These results indicate that the A3 analog forms the most stable complex with AF-COX-2 protein compared to all other analogs and controls. Furthermore, the SAS density, which is inversely proportional to the SASA, was determined to calculate the neighborhood density of burial HYD amino acids within the protein core that leads to protein folding^87^. The neighborhood density calculates the precise molecular and atomic quantities with coordinates for protein surface area accessible for solvent. It not only measures the hydrophobic effect of the amino acids density but also captures the electrostatic effect of the solvent on the protein folding and stability^88^. As demonstrated in Fig. 6e and Supplementary Table S9, the A3 analog possessed the least value of SAS density indicating better protein folding compared to other hit analogs and all controls after interaction with AF-COX-2 protein. All three solvent accessible surface analysis (area, volume, and density) together provide more emphasis on the MD simulation analysis related to the analogs’ complex stability in terms of protein folding and revealed more stability of analog A3 than the other studied compounds including the reference molecules. Overall, from MD simulation analysis, it can be inferred that A3 analog exhibit better protein stability than the other hit analogs, AGP, native protein, aspirin, and rofecoxib.

Moreover, the molecular interactions of the hit analogs were also analyzed after the MD simulation (Supplementary Fig. S3 and Table S10). Interestingly, except A7, the binding site for all the hit analogs were observed to be consistent before and after the simulation. Further, amino acid residues namely Val102, Arg106, Val335, Leu338, Ser339, Tyr341, Leu345, Phe504, Val509, Ala513, and Ser516 were found to be important residues before and after simulation as these are consistent before and after simulation. Further, a comparative analysis of before and after MD simulation in aspirin protein revealed that after simulation two hydrophobic interaction (Val509, Ala513) and two H-bonds (Arg106 and Arg499) are formed which were three and one respectively before the simulation. In case of rofecoxib, only three hydrophobic interactions (Leu338, Tyr371, and Trp373) were observed after the simulation, whereas before simulation one H-bond (residue) and six hydrophobic interactions (residue) were noted. Next, AGP protein complex exhibited six hydrophobic interactions without H-bond after simulation whereas three H-bond with six hydrophobic interaction were observed before simulation. Then, analog A1 protein complex showed two H-bonds with seven hydrophobic interactions after simulation while three H-bonds with five hydrophobic interactions were found before simulation. Analog A2 protein complex revealed nine hydrophobic interactions without any H-bonds after simulation whereas four H-bonds with six hydrophobic interactions were observed before simulation. Analog A3 protein complex displayed one H-bond with eight hydrophobic interactions after simulation while seven hydrophobic interactions along with one H-bond were observed before simulation. A4 protein complex exhibited one H-bond and seven hydrophobic interaction after simulation while three H-bonds with six hydrophobic interactions were observed before simulation. A5 protein complex revealed 2 H-bonds and seven hydrophobic interaction after simulation whereas 4 H-bonds with five hydrophobic interactions before simulation. A6 protein complex showed one H-bond with four hydrophobic interactions after simulation while only hydrophobic interactions were observed before simulation. Lastly, A7 protein complex revealed one H-bond with three hydrophobic interaction and only hydrophobic interactions were majorly involved before simulation (Supplementary Table S10). Overall, from the comparative analysis, it was observed that the hit analogs except the A7 remain close to the binding site identified by the molecular docking analysis which indicates the efficient binding stability of the complex of hit analogs with AF-COX-2. Also, analog A1, A3 and A5 complexes maintained their consistencies before and after simulation.

### Electrostatic potential energy simulation of the peptide-ligand complex

The EPE maps describe the distribution of electrons on the molecular surface, HOMO, and LUMO energy of the molecules. In this study, we have calculated the EPE, HOMO, and LUMO of the selected sequence of AF-COX-2 protein and its adduct with the AGP, hit AGP analogs, native protein, aspirin, and rofecoxib. Then, the binding energy of the adduct was computed, where the more negative value of binding energy indicates the more stability of the protein–ligand complex^89^. For this study, aspirin, rofecoxib, AGP, and A1–A7 AGP analogs were selected as ligands, while the protein sequences from 511 to 520 amino acids of AF-COX-2, i.e. Val511, Gly512, Ala513, Pro514, Phe515, Ser516, Leu517, Lys518, Gly519, Leu520 were selected as a peptide for docking simulation study by calculating EPE^35^. Later, the calculated binding energy values of the adducts were found to be in the range of − 53.81 kcal/mol (A3) to − 34.53 kcal/mol (aspirin) (Table 5). Further, all the AGP and all seven analogs exhibited lesser binding energy than the rofecoxib and aspirin, while the least binding energy value was observed for A3 suggesting the most stable protein–ligand complex.

**Table 5:** HOMO, LUMO, HLG, and electrostatic potential energy (EPE) of selected chemical compounds, AF-COX-2 peptide, and the adducts of the compounds with the AF-COX-2 peptide, as well as the binding energy for the complexes.

AF-COX-2 peptide / compound	HOMO (eV)	LUMO (eV)	HLG (eV)	EPE (kcal/mol) of compound	EPE (kcal/mol) of adduct	BE (kcal/mol) of (Adduct) = [Complex-(Peptide + Compound)]
Peptide	− 5.68	− 1.02	3.33	− 62.08	–	–
Rofecoxib	− 5.57	− 2.08	3.49	− 43.51	− 64.51	− 41.08
Aspirin	− 5.26	− 3.01	2.25	− 41.70	− 69.25	− 34.53
AGP	− 5.79	− 1.34	4.45	− 48.97	− 60.62	− 50.43
A1	− 5.85	− 1.04	4.81	− 45.97	− 60.05	− 48.00
A2	− 5.98	− 1.29	4.69	− 46.53	− 56.24	− 52.37
A3	− 5.77	− 1.18	4.59	− 48.31	− 56.58	− 53.81
A4	− 5.84	− 0.93	4.90	− 46.65	− 59.31	− 49.42
A5	− 5.62	− 1.20	4.42	− 43.98	− 63.88	− 42.18
A6	− 5.61	− 1.46	4.15	− 47.84	− 59.86	− 50.61
A7	− 5.59	− 1.19	4.40	− 48.98	− 63.63	− 47.43

HOMO,  highest occupied molecular orbital; LUMO,  lowest unoccupied molecular orbital; HLG,  HOMO–LUMO Gap; EPE, electrostatic potential energy, and BE,  binding energy.

### MM/GBSA rescoring analysis

**Figure 7:**
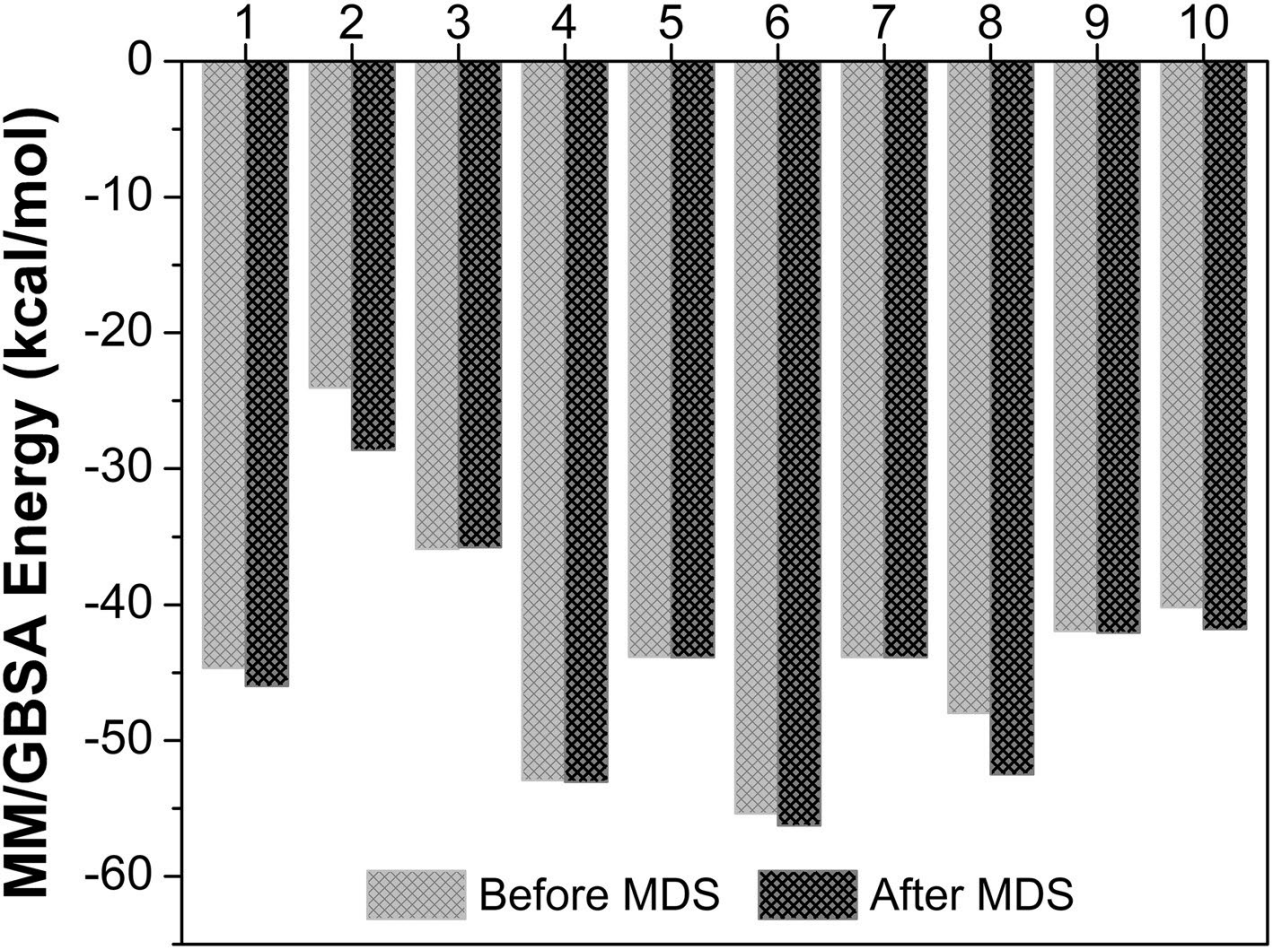
The MM/GBSA energy score of rofecoxib (1), aspirin (2), AGP (3), and selected analogs A1–A7 (4–10, respectively) for before and after MD simulation.

The binding free energy of the interacting hit AGP analogs, AGP, aspirin, and rofecoxib to the AF-COX-2 protein was computed by MM/GBSA method for before and after simulation analysis. The results revealed that there is a subtle difference between the MM/GBSA score for before and after simulation analysis (Fig. 7). Also, It can be noted from Fig. 7 that amongst all the investigated compounds, the A3 analog showed the most negative binding free energy (before simulation = − 55.37 kcal/mol and after simulation = − 56.25 kcal/mol) in terms of MM/GBSA indicating its strong interaction with AF-COX-2 protein. The MM/GBSA score was found to be in the decreasing order of binding affinity A3 > A1 > A5 > Rofecoxib > A2 > A4 > A6 > A7 > AGP > aspirin. Further, it was observed that all the hit AGP analogs along with the AGP exhibited a more negative value of free energy compared to aspirin, whereas analogs A3, A1, and A5 showed the binding affinity better than Rofecoxib, aspirin, AGP, and other analogs. Moreover, analog A3, A1 and A5 complex with protein were observed to be more stable after the simulation analysis and also implies that the analog A3 showed the better binding towards the COX-2 protein as compared to the reference compounds and other studied analogs. The MM/GBSA results also corroborated the findings of docking, MD simulation, and DFT analysis, and establish that the A3 analog would be a promising compound to inhibit the AF-COX-2 activity and can be used as a promising natural anti-inflammatory drug.

Overall, our results reveals that andrographolide analog A3 is the most potent and effective plant based natural drug molecule against COX-2 protein. The efficacy of this analog was compared with other natural compounds as well as synthetic compounds for COX-2 inhibition. The binding energy for A3 from docking analysis was found to be − 8.56 kcal/mol which is better than virgin coconut oil derivatives (BE range for the derivatives: − 5.65 kcal/mol to − 7.58 kcal/mol), alliin (− 4.90 kcal/mol), pinoresinolm (− 8.38 kcal/mol), and syringaresinol (− 8.23 kcal/mol)^90–93^. Further, MD simulation as well as EPE docking simulation analysis were carried for the complex stability for A3. Moreover, the detailed comparative analysis results with the reference compounds supports that the identified analog A3 would be an efficient drug-like candidate against COX-2 protein. To the best of our knowledge, to date, the andrographolide analog A3 has not been explored for anti-inflammatory activity by inhibiting the COX-2. Hence, present work provides a foundation for the development of andrographolide analog A3 as a potential anti-inflammatory drug-like candidate acting via COX-2 inhibition. Though, we have employed a myriad of cheminformatic tools for identification and validation of analog A3 as a potential COX-2 inhibitor, further experimental authentication is required from the scientific community to expedite the drug development process.

## Conclusion

In the present study, a promising plant-based anti-inflammatory compound against COX-2 was identified by employing a combined computational approach including molecular and quantum docking simulation studies. A validated full sequenced human AF-COX-2 protein structure was used for multiple sequence alignment to find out the active site residues for anti-inflammatory activity. The virtual screening of 237 AGP analogs against AF-COX-2 protein followed by interactive docking studies screened out 7 hit AGP analogs which showed their drug-like candidacy also from ADMET prediction analysis. Further, ligand efficiency metrics, DFT descriptors, i.e. HOMO, LUMO, HLG, and EPE established their good chemical reactivity. In addition, MD simulation, EPE docking simulation, and MM/GBSA study revealed that A3 analog (3-[2-[(1R,4aR,5R,6R,8aR)-6-hydroxy-5,6,8a-trimethyl-2-methylidene-3,4,4a,5,7,8-hexahydro-1H-naphthalen-1-yl]ethylidene]-4-hydroxyoxolan-2-one) forms the most stable complex with the AF-COX-2 and would be a promising natural anti-inflammatory agent. In near future, additional experimental validations are required to substantiate these findings.

## Supplementary Information

Supplementary Information.

## Data Availability

All data generated or analyzed during this study are included in this article and its Supplementary Information files. Additional raw data or datasets generated and/or analyzed during the current study are available from the corresponding author on reasonable request.

## References

[1] Smith WL, DeWitt DL, Garavito RM (2000). Cyclooxygenases: Structural, cellular, and molecular biology. Annu. Rev. Biochem..

[2] Vane JR (1971). Inhibition of prostaglandin synthesis as a mechanism of action for aspirin-like drugs. Nat. New Biol..

[3] Morita I (2002). Distinct functions of COX-1 and COX-2. Prostaglandins Other Lipid Mediat..

[4] Simon LS (1999). Role and regulation of cyclooxygenase-2 during inflammation. Am. J. Med..

[5] Orlando BJ, Malkowski MG (2016). Substrate-selective inhibition of cyclooxygeanse-2 by fenamic acid derivatives is dependent on peroxide tone. J. Biol. Chem..

[6] Rouzer CA, Marnett LJ (2003). Mechanism of free radical oxygenation of polyunsaturated fatty acids by cyclooxygenases. Chem. Rev..

[7] Wong E, Bayly C, Waterman HL, Riendeau D, Mancini JA (1997). Conversion of prostaglandin G/H synthase-1 into an enzyme sensitive to PGHS-2-selective inhibitors by a double His513→Arg and Ile523→Val mutation*. J. Biol. Chem..

[8] Gierse JK (1996). A single amino acid difference between cyclooxygenase-1 (COX-1) and −2 (COX-2) reverses the selectivity of COX-2 specific inhibitors*. J. Biol. Chem..

[9] Cipollone F, Cicolini G, Bucci M (2008). Cyclooxygenase and prostaglandin synthases in atherosclerosis: Recent insights and future perspectives. Pharmacol. Ther..

[10] Rouzer CA, Marnett LJ (2009). Cyclooxygenases: Structural and functional insights. J. Lipid Res..

[11] Blobaum AL, Marnett LJ (2007). Structural and functional basis of cyclooxygenase inhibition. J. Med. Chem..

[12] Wallace JL (2002). Selective cyclooxygenase-2 inhibitors: After the smoke has cleared. Dig. Liver Dis..

[13] Marnett LJ (2009). The COXIB experience: A look in the rearview mirror. Annu. Rev. Pharmacol. Toxicol..

[14] Hilário MOE, Terreri MT, Len CA (2006). Nonsteroidal anti-inflammatory drugs: Cyclooxygenase 2 inhibitors. J. Pediatr. (Rio. J.).

[15] Ahuja N, Singh A, Singh B (2010). Rofecoxib: An update on physicochemical, pharmaceutical, pharmacodynamic and pharmacokinetic aspects. J. Pharm. Pharmacol..

[16] Mukherjee D, Nissen SE, Topol EJ (2001). Risk of cardiovascular events associated with selective COX-2 inhibitors. JAMA.

[17] Funk CD, FitzGerald GA (2007). COX-2 inhibitors and cardiovascular risk. J. Cardiovasc. Pharmacol..

[18] Chen W (2021). New horizons in the roles and associations of COX-2 and novel natural inhibitors in cardiovascular diseases. Mol. Med..

[19] Andersohn F, Schade R, Suissa S, Garbe E (2006). Cyclooxygenase-2 selective nonsteroidal anti-inflammatory drugs and the risk of ischemic stroke: A nested case-control study. Stroke.

[20] Attiq A, Jalil J, Husain K, Ahmad W (2018). Raging the war against inflammation with natural products. Front. Pharmacol..

[21] Lee KC, Chang HH, Chung YH, Lee TY (2011). Andrographolide acts as an anti-inflammatory agent in LPS-stimulated RAW264.7 macrophages by inhibiting STAT3-mediated suppression of the NF-κB pathway. J. Ethnopharmacol..

[22] Yuan L (2016). The semi-synthesis of novel andrographolide analogues and anti-influenza virus activity evaluation of their derivatives. Bioorganic Med. Chem. Lett..

[23] Jiao J (2019). Screening cyclooxygenase-2 inhibitors from *Andrographis*
*paniculata* to treat inflammation based on bio-affinity ultrafiltration coupled with UPLC-Q-TOF-MS. Fitoterapia.

[24] Singh J, Petter RC, Baillie TA, Whitty A (2011). The resurgence of covalent drugs. Nat. Rev. Drug Discov..

[25] Tran QTN, Tan DWS, Wong WSF, Chai CLL (2020). From irreversible to reversible covalent inhibitors: Harnessing the andrographolide scaffold for anti-inflammatory action. Eur. J. Med. Chem..

[26] Tran QTN, Tan WSD, Wong WSF, Chai CLL (2021). Polypharmacology of andrographolide: Beyond one molecule one target. Nat. Prod. Rep..

[27] Nguyen VS (2015). Specificity and inhibitory mechanism of andrographolide and its analogues as antiasthma agents on NF-κB p50. J. Nat. Prod..

[28] Burgos RA, Alarcón P, Quiroga J, Manosalva C, Hancke J (2021). Andrographolide, an anti-inflammatory multitarget drug: All roads lead to cellular metabolism. Molecules.

[29] Dai GF (2011). Anti-inflammatory effect of novel andrographolide derivatives through inhibition of NO and PGE 2 production. Int. Immunopharmacol..

[30] Wang W (2019). Synthesis of new ent-labdane diterpene derivatives from andrographolide and evaluation of their anti-inflammatory activities. Eur. J. Med. Chem..

[31] Peng Y (2018). Andrographolide inhibits breast cancer through suppressing COX-2 expression and angiogenesis via inactivation of p300 signaling and VEGF pathway 11 medical and health sciences 1112 oncology and carcinogenesis. J. Exp. Clin. Cancer Res..

[32] Liu W (2020). Andrographolide potentiates PD-1 blockade immunotherapy by inhibiting COX2-mediated PGE2 release. Int. Immunopharmacol..

[33] Chen M, Xie C, Liu L (2010). Solubility of andrographolide in various solvents from (288.2 to 323.2) K. J. Chem. Eng. Data.

[34] Tunyasuvunakool K (2021). Highly accurate protein structure prediction for the human proteome. Nature.

[35] Lucido MJ, Orlando BJ, Vecchio AJ, Malkowski MG (2016). Crystal structure of aspirin–acetylated human cyclooxygenase-2: Insight into the formation of products with reversed stereochemistry. Biochemistry.

[36] Orlando BJ, Malkowski MG (2016). Crystal structure of rofecoxib bound to human cyclooxygenase-2. Acta Crystallogr. Sect. Struct. Biol. Commun..

[37] Orlando BJ, Malkowski MG (2016). Substrate-selective Inhibition of cyclooxygeanse-2 by fenamic acid derivatives is dependent on peroxide tone*. J. Biol. Chem..

[38] Colovos C, Yeates TO (1993). Verification of protein structures: Patterns of nonbonded atomic interactions. Protein Sci..

[39] Pontius J, Richelle J, Wodak SJ (1996). Deviations from standard atomic volumes as a quality measure for protein crystal structures. J. Mol. Biol..

[40] Wiederstein M, Sippl MJ (2007). ProSA-web: Interactive web service for the recognition of errors in three-dimensional structures of proteins. Nucleic Acids Res..

[41] Wallner B, Elofsson A (2003). Can correct protein models be identified?. Protein Sci..

[42] Cristobal S, Zemla A, Fischer D, Rychlewski L, Elofsson A (2001). A study of quality measures for protein threading models. BMC Bioinformatics.

[43] Bhattacharya D, Nowotny J, Cao R, Cheng J (2016). 3Drefine: An interactive web server for efficient protein structure refinement. Nucleic Acids Res..

[44] Tian W, Chen C, Lei X, Zhao J, Liang J (2018). CASTp 3.0: computed atlas of surface topography of proteins. Nucleic Acids Res..

[45] Pettersen EF (2004). UCSF Chimera—A visualization system for exploratory research and analysis. J. Comput. Chem..

[46] Simossis VA, Heringa J (2005). PRALINE: A multiple sequence alignment toolbox that integrates homology-extended and secondary structure information. Nucleic Acids Res..

[47] Agarwal S, Dixit A, Kashaw SK (2020). Ligand and structure based virtual screening of chemical databases to explore potent small molecule inhibitors against breast invasive carcinoma using recent computational technologies. J. Mol. Graph. Model..

[48] Kim S (2021). PubChem in 2021: New data content and improved web interfaces. Nucleic Acids Res..

[49] O’Boyle NM (2011). Open babel: An open chemical toolbox. J. Cheminform..

[50] Dallakyan S, Olson AJ, Hempel JE, Williams CH, Hong CC (2015). Small-molecule library screening by docking with PyRx. Chemical biology: Methods and protocols.

[51] Morris GM (2009). AutoDock4 and AutoDockTools4: Automated docking with selective receptor flexibility. J. Comput. Chem..

[52] Abagyan R, Totrov M, Kuznetsov D (1994). ICM—A new method for protein modeling and design: Applications to docking and structure prediction from the distorted native conformation. J. Comput. Chem..

[53] Xiong G (2021). ADMETlab 2.0: An integrated online platform for accurate and comprehensive predictions of ADMET properties. Nucleic Acids Res..

[54] Hopkins AL, Keserü GM, Leeson PD, Rees DC, Reynolds CH (2014). The role of ligand efficiency metrics in drug discovery. Nat. Rev. Drug Discov..

[55] Onawole AT, Kolapo TU, Sulaiman KO, Adegoke RO (2018). Structure based virtual screening of the Ebola virus trimeric glycoprotein using consensus scoring. Comput. Biol. Chem..

[56] Schultes S (2010). Ligand efficiency as a guide in fragment hit selection and optimization. Drug Discov. Today Technol..

[57] Murray CW (2014). Validity of ligand efficiency metrics. ACS Med. Chem. Lett..

[58] Reynolds CH, Bembenek SD, Tounge BA (2007). The role of molecular size in ligand efficiency. Bioorg. Med. Chem. Lett..

[59] Becke AD (1993). Density-functional thermochemistry. III: The role of exact exchange. J. Chem. Phys..

[60] Rassolov VA, Ratner MA, Pople JA, Redfern PC, Curtiss LA (2001). 6–31G* basis set for third-row atoms. J. Comput. Chem..

[61] Gill PMW, Johnson BG, Pople JA, Frisch MJ (1992). The performance of the Becke–Lee–Yang–Parr (B–LYP) density functional theory with various basis sets. Chem. Phys. Lett..

[62] Stephens PJ, Devlin FJ, Chabalowski CF, Frisch MJ (1994). Ab Initio calculation of vibrational absorption and circular dichroism spectra using density functional force fields. J. Phys. Chem..

[63] Abraham MJ (2015). Gromacs: High performance molecular simulations through multi-level parallelism from laptops to supercomputers. SoftwareX.

[64] Schüttelkopf AW, Van Aalten DMF (2004). PRODRG: A tool for high-throughput crystallography of protein-ligand complexes. Acta Crystallogr. Sect. D Biol. Crystallogr..

[65] Schmid N (2011). Definition and testing of the GROMOS force-field versions 54A7 and 54B7. Eur. Biophys. J..

[66] Abdel-Mottaleb MSA, Abdel-Mottaleb Y (2021). Impact of magnesium, zinc, selenium, copper, and iodine food supplements on SARS-CoV, SARS-CoV-2 viruses and their adducts with human ACE2 enzyme: A Computational Based Investigation. Egypt. J. Chem..

[67] Wang Z (2019). FarPPI: A webserver for accurate prediction of protein-ligand binding structures for small-molecule PPI inhibitors by MM/PB(GB)SA methods. Bioinformatics.

[68] Maier JA (2015). ff14SB: Improving the accuracy of protein side chain and backbone parameters from ff99SB. J. Chem. Theory Comput..

[69] Jakalian A, Jack DB, Bayly CI (2002). Fast, efficient generation of high-quality atomic charges. AM1-BCC model: II. Parameterization and validation. J. Comput. Chem..

[70] Wang E (2019). End-point binding free energy calculation with MM/PBSA and MM/GBSA: Strategies and applications in drug design. Chem. Rev..

[71] Liang J, Woodward C, Edelsbrunner H (1998). Anatomy of protein pockets and cavities: Measurement of binding site geometry and implications for ligand design. Protein Sci..

[72] Banavath HN, Sharma OP, Kumar MS, Baskaran R (2014). Identification of novel tyrosine kinase inhibitors for drug resistant T315I mutant BCR–ABL: A virtual screening and molecular dynamics simulations study. Sci. Rep..

[73] Palaka BK, Venkatesan R, Ampasala DR, Periyasamy L (2019). Identification of novel inhibitors of signal transducer and activator of transcription 3 over signal transducer and activator of transcription 1 for the treatment of breast cancer by in-silico and in-vitro approach. Process Biochem..

[74] Chaudhary MK (2013). Computational evaluation on molecular stability, reactivity, and drug potential of frovatriptan from DFT and molecular docking approach. Comput. Theor. Chem..

[75] Noureddine O, Issaoui N, Al-Dossary O (2021). DFT and molecular docking study of chloroquine derivatives as antiviral to coronavirus COVID-19. J. King Saud Univ. Sci..

[76] Mottishaw JD, Erck AR, Kramer JH, Sun H, Koppang M (2015). Electrostatic potential maps and natural bond orbital analysis: Visualization and conceptualization of reactivity in Sangers reagent. J. Chem. Educ..

[77] Uzzaman M, Junaid M, Uddin MN (2020). Evaluation of anti-tuberculosis activity of some oxotitanium(IV) Schiff base complexes; molecular docking, dynamics simulation and ADMET studies. SN Appl. Sci..

[78] Horton DA, Bourne GT, Smythe ML (2003). The combinatorial synthesis of bicyclic privileged structures or privileged substructures. Chem. Rev..

[79] Hajduk PJ, Bures M, Praestgaard J, Fesik SW (2000). Privileged molecules for protein binding identified from NMR-based screening. J. Med. Chem..

[80] El-Haj BM, Ahmed SBM (2020). Metabolic-hydroxy and carboxy functionalization of alkyl moieties in drug molecules: Prediction of structure influence and pharmacologic activity. Molecules.

[81] Unal MA, Boyacioglu B, Unver H, Elmali A (2019). Molecular simulation of PcCel45A protein expressed from *Aspergillus*
*nidulans* to understand its structure, dynamics, and thermostability. J. Mol. Model..

[82] Lobanov MY, Bogatyreva NS, Galzitskaya OV (2008). Radius of gyration as an indicator of protein structure compactness. Mol. Biol..

[83] Chen CR, Makhatadze GI (2015). ProteinVolume: Calculating molecular van der Waals and void volumes in proteins. BMC Bioinformatics.

[84] Kim IJ, Na H (2022). An efficient algorithm calculating common solvent accessible volume. PLoS ONE.

[85] Chen CR, Makhatadze GI (2015). ProteinVolume: Calculating molecular van der Waals and void volumes in proteins. BMC Bioinformatics.

[86] Wagoner JA, Baker NA (2006). Assessing implicit models for nonpolar mean solvation forces: The importance of dispersion and volume terms. Proc. Natl. Acad. Sci..

[87] Durham E, Dorr B, Woetzel N, Staritzbichler R, Meiler J (2009). Solvent accessible surface area approximations for rapid and accurate protein structure prediction. J. Mol. Model..

[88] Weiser J, Shenkin PS, Still WC (1999). Approximate solvent–Accessible surface areas from tetrahedrally directed neighbor densities. Biopolymers.

[89] Abdel-Mottaleb MSA, Abdel-Mottaleb Y (2020). In search for effective and safe drugs against SARS-CoV-2: Part I] simulated interactions between selected nutraceuticals, ACE2 enzyme and S Protein simple peptide sequences. ChemRxiv.

[90] Taidi L, Maurady A, Britel MR (2022). Molecular docking study and molecular dynamic simulation of human cyclooxygenase-2 (COX-2) with selected eutypoids. J. Biomol. Struct. Dyn..

[91] Dhanjal JK (2015). Computational structure-based de novo design of hypothetical inhibitors against the anti-inflammatory target COX-2. PLoS ONE.

[92] Jack KS, Asaruddin MRB, Bhawani SA (2022). Pharmacophore study, molecular docking and molecular dynamic simulation of virgin coconut oil derivatives as anti-inflammatory agent against COX-2. Chem. Biol. Technol. Agric..

[93] Sadeghi M, Miroliaei M, Fateminasab F, Moradi M (2021). Screening cyclooxygenase-2 inhibitors from *Allium sativum* L. compounds: In silico approach. J. Mol. Model..

