# Supplementary material for: Discovery of andrographolide hit analog as a potent cyclooxygenase-2 inhibitor through consensus MD-simulation, electrostatic potential energy simulation and ligand efficiency metrics
Source: Sci Rep. 2023 May 19;13:8147. doi: 10.1038/s41598-023-35192-7 (PMC10199084; doi:10.1038/s41598-023-35192-7)
Supplement: Supplementary file 1 — Supplementary Information. [file 41598_2023_35192_MOESM1_ESM.docx]

**Supplementary Information For**

**Discovery of andrographolide hit analog as a potent cyclooxygenase-2 inhibitor through consensus MD-simulation, electrostatic potential energy simulation and ligand efficiency metrics**

Priyanka Jain^1^, Jitendra Satija^2^ and C. Sudandiradoss^1*^

^1^ School of Biosciences and Technology, Vellore Institute of Technology, Vellore, Tamil Nadu 632014, India

^2^ Centre for Nanobiotechnology, Vellore Institute of Technology, Vellore, Tamil Nadu 632014, India

* Corresponding Author: Prof. C. Sudandiradoss (csudandiradoss@vit.ac.in; franklindoss@gmail.com)

**Supplementary Table S1.** Validation results of AF-COX-2 protein.

| **Protein** | **Method** | **Resolution** | **Chain** | **AA length** | **ERRAT** | **VERIFY** | **Ramachandran plot**  **(**Residue in most favored reason) | **ProSA**  **(Z score)** | **ProQ**  **LGscore** |
| --- | --- | --- | --- | --- | --- | --- | --- | --- | --- |
| 5F19 | X-ray | 2.04 | A/B | 19-569 | 89.61 | Pass | 92.8 | -8.91 | 7.617 |
| 5KIR | X-ray | 2.70 | A/B | 19-569 | 88.08 | Pass | 92.2 | -8.82 | -0.835 |
| 5F1A | X-ray | 2.38 | A/B | 19-570 | 97.32 | Fail | 89.5 | -8.59 | 7.593 |
| 5IKQ | X-ray | 2.41 | A/B | 19-569 | 96.31 | Fail | 90.2 | -8.73 | -0.835 |
| 1V0X | predicted | - | - | 1-604 | 83.19 | Pass | 90.8 | -7.69 | 5.749 |
| AF-P35354-F1 | Predicted | - | - | 1-604 | 97.74 | Pass | 90.9 | -8.97 | 7.639 |

**Supplementary Table S2**. CASTp results for AF-COX-2 protein for binding pocket prediction

| **Pocket ID** | **N_mth** | **Area_sa** | **Area_ms** | **Vol_sa** | **Vol_ms** | **Lenth** | **cnr** |
| --- | --- | --- | --- | --- | --- | --- | --- |
| 1 | 7 | 1561.244 | 2325.755 | 1335.282 | 3960.627 | 1178.503 | 527 |
| **2*** | **10** | **1485.989** | **2465.405** | **902.549** | **3591.843** | **1245.454** | **552** |
| 3 | 1 | 237.117 | 314.506 | 315.052 | 703.055 | 180.259 | 70 |
| 4 | 0 | 119.339 | 319.44 | 43.724 | 336.963 | 144.149 | 88 |
| 5 | 3 | 87.474 | 191.386 | 38.272 | 222.014 | 105.285 | 51 |
| 6 | 1 | 111.976 | 359.266 | 27.652 | 327.718 | 153.655 | 84 |
| 7 | 1 | 26.975 | 54.724 | 26.118 | 75.959 | 21.042 | 18 |
| 8 | 1 | 32.977 | 69.936 | 22.936 | 94.256 | 40.316 | 22 |
| 9 | 0 | 87.538 | 281.929 | 22.89 | 266.299 | 110.648 | 74 |
| 10 | 1 | 42.117 | 100.223 | 20.669 | 112.142 | 42.902 | 22 |
| 11 | 1 | 42.403 | 97.624 | 19.096 | 115.085 | 52.059 | 30 |
| 12 | 1 | 24.291 | 52.841 | 10.233 | 60.481 | 24.498 | 20 |
| 13 | 1 | 23.726 | 67.835 | 9.635 | 71.388 | 33.542 | 21 |
| 14 | 1 | 23.196 | 59.507 | 9.453 | 66.121 | 29.452 | 14 |
| 15 | 2 | 52.69 | 173.169 | 9.216 | 160.033 | 78.567 | 44 |
| 16 | 1 | 38.394 | 117.435 | 8.99 | 116.231 | 64.126 | 44 |
| 17 | 1 | 42.649 | 154.167 | 8.894 | 140.967 | 69.405 | 46 |
| 18 | 0 | 30.142 | 132.525 | 5.727 | 111.843 | 54.075 | 42 |
| 19 | 1 | 23.226 | 78.953 | 5.084 | 70.657 | 30.457 | 25 |
| 20 | 0 | 32.912 | 185.778 | 4.896 | 146.519 | 71.723 | 52 |
| 21 | 1 | 18.363 | 69.784 | 4.822 | 63.365 | 32.684 | 24 |
| 22 | 1 | 22 | 71.061 | 4.644 | 61.215 | 23.464 | 16 |
| 23 | 1 | 11.034 | 20.054 | 4.338 | 25.216 | 8.943 | 5 |
| 24 | 1 | 18.759 | 66.716 | 4.002 | 58.37 | 27.049 | 24 |
| 25 | 0 | 17.995 | 103.164 | 3.792 | 81.249 | 36.726 | 34 |
| 26 | 1 | 14.372 | 41.117 | 3.622 | 41.882 | 25.041 | 18 |
| 27 | 0 | 19.36 | 105.441 | 3.398 | 84.77 | 36.705 | 24 |
| 28 | 1 | 10.014 | 35.412 | 3.326 | 34.171 | 17.572 | 13 |
| 29 | 1 | 15.283 | 56.995 | 2.592 | 44.849 | 23.858 | 18 |
| 30 | 1 | 10.742 | 49.532 | 2.057 | 39.813 | 18.733 | 16 |
| 31 | 0 | 13.406 | 126.886 | 1.773 | 81.223 | 37.418 | 28 |
| 32 | 1 | 11.996 | 76.779 | 1.698 | 59.678 | 31.504 | 33 |
| 33 | 0 | 13.4 | 122.165 | 1.326 | 76.084 | 32.592 | 28 |
| 34 | 1 | 8.002 | 49.295 | 1.233 | 37.988 | 17.618 | 16 |
| 35 | 0 | 8.778 | 74.394 | 1.22 | 51.562 | 23.573 | 20 |
| 36 | 1 | 3.982 | 23.235 | 1.014 | 18.424 | 9.327 | 9 |
| 37 | 1 | 9.007 | 108.007 | 0.778 | 53.516 | 30.548 | 24 |
| 38 | 0 | 6.536 | 67.974 | 0.707 | 46.029 | 19.083 | 20 |
| 39 | 1 | 5.231 | 44.334 | 0.616 | 30.67 | 14.674 | 17 |
| 40 | 0 | 5.276 | 58.503 | 0.559 | 39.287 | 16.908 | 18 |
| 41 | 1 | 4.065 | 22.518 | 0.521 | 16.664 | 6.595 | 8 |
| 42 | 2 | 4.349 | 22.5 | 0.496 | 19.756 | 12.158 | 10 |
| 43 | 1 | 4.825 | 38.478 | 0.481 | 26.798 | 12.353 | 12 |
| 44 | 1 | 3.303 | 28.878 | 0.471 | 20.498 | 10.518 | 10 |
| 45 | 2 | 5.557 | 36.499 | 0.466 | 28.096 | 15.531 | 11 |
| 46 | 0 | 4.48 | 60.36 | 0.432 | 37.846 | 15.761 | 16 |
| 47 | 0 | 3.7 | 58.054 | 0.253 | 35.782 | 12.658 | 14 |
| 48 | 0 | 3.854 | 66.922 | 0.249 | 35.056 | 12.721 | 14 |
| 49 | 0 | 3.731 | 62.881 | 0.248 | 36.004 | 12.814 | 16 |
| 50 | 0 | 3.073 | 53.241 | 0.227 | 31.055 | 11.316 | 16 |
| 51 | 1 | 1.919 | 23.275 | 0.217 | 16.41 | 6.443 | 6 |
| 52 | 1 | 1.292 | 7.754 | 0.214 | 6.156 | 3.111 | 3 |
| 53 | 0 | 2.915 | 65.329 | 0.154 | 40.686 | 14.623 | 20 |
| 54 | 1 | 1.323 | 12.105 | 0.151 | 8.257 | 3.895 | 5 |
| 55 | 0 | 2.193 | 50.124 | 0.144 | 27.388 | 8.969 | 10 |
| 56 | 0 | 2.493 | 75.692 | 0.134 | 35.582 | 13.757 | 16 |
| 57 | 0 | 2.66 | 51.109 | 0.128 | 31.049 | 11.531 | 14 |
| 58 | 0 | 2.076 | 45.815 | 0.12 | 27.272 | 8.105 | 10 |
| 59 | 0 | 2.507 | 61.298 | 0.119 | 33.031 | 10.671 | 12 |
| 60 | 0 | 3.924 | 87.196 | 0.108 | 32.479 | 17.158 | 18 |
| 61 | 0 | 2.101 | 50.104 | 0.089 | 29.766 | 9.689 | 14 |
| 62 | 1 | 0.934 | 13.966 | 0.085 | 8.932 | 3.095 | 4 |
| 63 | 0 | 1.491 | 43.065 | 0.084 | 24.897 | 6.489 | 8 |
| 64 | 0 | 1.443 | 43.054 | 0.065 | 25.047 | 6.823 | 8 |
| 65 | 0 | 1.171 | 41.051 | 0.055 | 23.156 | 6.05 | 10 |
| 66 | 1 | 0.609 | 10.138 | 0.041 | 5.976 | 1.939 | 2 |
| 67 | 1 | 0.661 | 20.61 | 0.039 | 12.069 | 4.543 | 6 |
| 68 | 0 | 0.965 | 43.487 | 0.029 | 23.151 | 5.817 | 8 |
| 69 | 0 | 0.876 | 44.937 | 0.026 | 24.887 | 7.527 | 8 |
| 70 | 0 | 0.783 | 43.694 | 0.025 | 22.27 | 5.562 | 8 |
| 71 | 0 | 0.966 | 55.278 | 0.021 | 26.994 | 8.201 | 14 |
| 72 | 0 | 0.434 | 33.535 | 0.013 | 17.828 | 3.302 | 8 |
| 73 | 0 | 0.448 | 35.845 | 0.012 | 19.235 | 4.63 | 8 |
| 74 | 0 | 0.47 | 37.22 | 0.01 | 19.409 | 4.065 | 6 |
| 75 | 1 | 0.251 | 16.917 | 0.009 | 9.114 | 1.983 | 4 |
| 76 | 0 | 0.22 | 30.617 | 0.005 | 15.839 | 2.646 | 10 |
| 77 | 0 | 0.168 | 30.218 | 0.003 | 15.51 | 2.027 | 6 |
| 78 | 0 | 0.175 | 30.766 | 0.003 | 15.869 | 2.193 | 4 |
| 79 | 0 | 0.134 | 29.015 | 0.003 | 14.61 | 1.942 | 10 |
| 80 | 0 | 0.181 | 30.772 | 0.003 | 15.689 | 2.153 | 6 |
| 81 | 0 | 0.227 | 38.2 | 0.002 | 17.282 | 3.4 | 14 |
| 82 | 0 | 0.116 | 31.01 | 0.001 | 15.626 | 2.458 | 8 |
| 83 | 0 | 0.068 | 27.837 | 0.001 | 13.786 | 1.289 | 6 |
| 84 | 0 | 0.1 | 29.674 | 0.001 | 14.735 | 1.646 | 6 |
| 85 | 0 | 0.092 | 28.788 | 0.001 | 14.47 | 1.506 | 4 |
| 86 | 0 | 0.004 | 25.627 | 0 | 12.188 | 0.366 | 6 |
| 87 | 0 | 0.024 | 26.608 | 0 | 12.885 | 0.848 | 6 |
| 88 | 0 | 0.019 | 26.476 | 0 | 12.747 | 0.688 | 6 |
| 89 | 0 | 0.002 | 25.185 | 0 | 11.884 | 0.207 | 4 |
| 90 | 0 | 0.02 | 26.355 | 0 | 12.714 | 0.726 | 6 |
| 91 | 1 | 0.027 | 19.77 | 0 | 6.856 | 0.633 | 3 |
| 92 | 0 | 0.001 | 25.327 | 0 | 11.759 | 0.142 | 4 |
| 93 | 0 | 0.02 | 28.068 | 0 | 13.093 | 0.853 | 4 |
| 94 | 0 | 0.041 | 29.765 | 0 | 13.899 | 1.332 | 8 |
| 95 | 0 | 0.005 | 25.665 | 0 | 12.207 | 0.366 | 4 |
| 96 | 0 | 0.056 | 30.225 | 0 | 14.153 | 1.347 | 4 |
| Footnote: N_mth= number of mouths, or opening to the external molecular surface; Area_sa= pocket solvent-accessible surface area; Area_ms= pocket molecular surface area; Vol_sa= pocket volume based on the solvent-accessible surface; Vol_ms= pocket volume based on the molecular surface; sum arc Length= total length of arcs formed where two pocket atoms meet; Corner points= number of corner points formed where three pocket atoms meet.  *Amino acids present in this pocket: His75, Arg106, Phe191, Val214, Val330, Ile331, Tyr334, Val335, Leu338, Tyr341, Leu345, Lys346, Gln358, Asn361, Tyr371, Trp373, Lys454, Arg455, Met457, Arg499, Phe504, Glu510, Phe515, Ser516, Leu517, Leu520, Met521. | | | | | | | |

**Supplementary Table S3.** Multiple sequence alignment results.

| **S. No.** | **Parameter** | **Score** |
| --- | --- | --- |
|  | Alignment score | 476382.00 |
|  | Alignment score per aligned residue pair | 17.54 |
|  | Sequence identities | 23637 |
|  | Percent sequence identity | 0.87 |
|  | Number of sequences | 10 |
|  | Alignment length | 604 |
|  | Number of residues | 6038 |
|  | Number of gaps | 2 |

**Supplementary Table S4.** Virtual Screening of 237 AGP analogs against AF-COX-2 protein.

| **S. No.** | **Analogs PubChem ID** | **Binding Affinity (kcal/mol)** |
| --- | --- | --- |
|  | 132210370 | -8.8 |
|  | 59876529 | -8.6 |
|  | 132210549 | -8.5 |
|  | 46871816 | -8.4 |
|  | 71550939 | -8.4 |
|  | 59876521 | -8.3 |
|  | 59876523 | -8.3 |
|  | 132210372 | -8.2 |
|  | 132210388 | -8.2 |
|  | 132210478 | -8.2 |
|  | 132210508 | -8.2 |
|  | 132217538 | -8.2 |
|  | 10041596 | -8.1 |
|  | 127040428 | -8.1 |
|  | 127042992 | -8.1 |
|  | 129317078 | -8.1 |
|  | 132210396 | -8.1 |
|  | 132210409 | -8.1 |
|  | 132210515 | -8.1 |
|  | 16122395 | -8.1 |
|  | 44409328 | -8.1 |
|  | 44574537 | -8.1 |
|  | 10567584 | -8 |
|  | 127042688 | -8 |
|  | 132210365 | -8 |
|  | 132210373 | -8 |
|  | 132210390 | -8 |
|  | 59876519 | -8 |
|  | 10065491 | -7.9 |
|  | 127040078 | -7.9 |
|  | 132210516 | -7.9 |
|  | 44411126 | -7.9 |
|  | 69649697 | -7.9 |
|  | 102463322 | -7.8 |
|  | 127040079 | -7.8 |
|  | 132210395 | -7.8 |
|  | 132217527 | -7.8 |
|  | 132217578 | -7.8 |
|  | 44411016 | -7.8 |
|  | 101362374 | -7.7 |
|  | 132210552 | -7.7 |
|  | 132210553 | -7.7 |
|  | 132217489 | -7.7 |
|  | 44411130 | -7.7 |
|  | 46906680 | -7.7 |
|  | 59921241 | -7.7 |
|  | 71578897 | -7.7 |
|  | 72191643 | -7.7 |
|  | 102007111 | -7.6 |
|  | 132210481 | -7.6 |
|  | 132223154 | -7.6 |
|  | 132223164 | -7.6 |
|  | 44409284 | -7.6 |
|  | 44409292 | -7.6 |
|  | 59876516 | -7.6 |
|  | 132210368 | -7.5 |
|  | 132210408 | -7.5 |
|  | 132217499 | -7.5 |
|  | 132217521 | -7.5 |
|  | 132217570 | -7.5 |
|  | 16038718 | -7.5 |
|  | 59876491 | -7.5 |
|  | 132210482 | -7.4 |
|  | 132217478 | -7.4 |
|  | 132217497 | -7.4 |
|  | 132223040 | -7.4 |
|  | 59070397 | -7.4 |
|  | 59143923 | -7.4 |
|  | 59876534 | -7.4 |
|  | 68169653 | -7.4 |
|  | 90710032 | -7.4 |
|  | 90922728 | -7.4 |
|  | 92215519 | -7.4 |
|  | 123361152 | -7.3 |
|  | 132210355 | -7.3 |
|  | 132210479 | -7.3 |
|  | 132217514 | -7.3 |
|  | 132217515 | -7.3 |
|  | 132217529 | -7.3 |
|  | 132223170 | -7.3 |
|  | 24980082 | -7.3 |
|  | 46906151 | -7.3 |
|  | 58209855 | -7.3 |
|  | 90783400 | -7.3 |
|  | 91032405 | -7.3 |
|  | 102463323 | -7.2 |
|  | 132210460 | -7.2 |
|  | 132210477 | -7.2 |
|  | 132210507 | -7.2 |
|  | 132217516 | -7.2 |
|  | 132217523 | -7.2 |
|  | 132223153 | -7.2 |
|  | 44393859 | -7.2 |
|  | 44411049 | -7.2 |
|  | 50908714 | -7.2 |
|  | 57395012 | -7.2 |
|  | 59876511 | -7.2 |
|  | 132210364 | -7.1 |
|  | 132210480 | -7.1 |
|  | 132217511 | -7.1 |
|  | 132217520 | -7.1 |
|  | 132217526 | -7.1 |
|  | 44575278 | -7.1 |
|  | 44575279 | -7.1 |
|  | 46907192 | -7.1 |
|  | 57391527 | -7.1 |
|  | 59876514 | -7.1 |
|  | 59897203 | -7.1 |
|  | 71590622 | -7.1 |
|  | 86578936 | -7.1 |
|  | 89809110 | -7.1 |
|  | 10914618 | -7 |
|  | 11624161 | -7 |
|  | 130324504 | -7 |
|  | 132210461 | -7 |
|  | 132217517 | -7 |
|  | 16758033 | -7 |
|  | 23757051 | -7 |
|  | 57403747 | -7 |
|  | 59741079 | -7 |
|  | 59897206 | -7 |
|  | 73357050 | -7 |
|  | 91408358 | -7 |
|  | 118566340 | -6.9 |
|  | 132223104 | -6.9 |
|  | 15922990 | -6.9 |
|  | 24879275 | -6.9 |
|  | 24879276 | -6.9 |
|  | 25114469 | -6.9 |
|  | 452937 | -6.9 |
|  | 46907311 | -6.9 |
|  | 5318517 | -6.9 |
|  | 59070369 | -6.9 |
|  | 59453160 | -6.9 |
|  | 59876490 | -6.9 |
|  | 59876507 | -6.9 |
|  | 6436016 | -6.9 |
|  | 6473762 | -6.9 |
|  | 6708647 | -6.9 |
|  | 6857767 | -6.9 |
|  | 7061132 | -6.9 |
|  | 71307452 | -6.9 |
|  | 71624124 | -6.9 |
|  | 71624251 | -6.9 |
|  | 91268827 | -6.9 |
|  | 91499723 | -6.9 |
|  | 11078630 | -6.8 |
|  | 11869597 | -6.8 |
|  | 118712674 | -6.8 |
|  | 118712675 | -6.8 |
|  | 118712676 | -6.8 |
|  | 123516411 | -6.8 |
|  | 132210551 | -6.8 |
|  | 20054828 | -6.8 |
|  | 44575273 | -6.8 |
|  | 46907271 | -6.8 |
|  | 5708351 | -6.8 |
|  | 57395013 | -6.8 |
|  | 57401998 | -6.8 |
|  | 59070294 | -6.8 |
|  | 59070301 | -6.8 |
|  | 59741104 | -6.8 |
|  | 59897198 | -6.8 |
|  | 7061134 | -6.8 |
|  | 7067324 | -6.8 |
|  | 73353957 | -6.8 |
|  | 91221436 | -6.8 |
|  | 123179837 | -6.7 |
|  | 123430276 | -6.7 |
|  | 135339861 | -6.7 |
|  | 16394566 | -6.7 |
|  | 16406675 | -6.7 |
|  | 23757050 | -6.7 |
|  | 25114900 | -6.7 |
|  | 38350572 | -6.7 |
|  | 44393988 | -6.7 |
|  | 46907313 | -6.7 |
|  | 59070286 | -6.7 |
|  | 65153 | -6.7 |
|  | 68103125 | -6.7 |
|  | 73351017 | -6.7 |
|  | 90765060 | -6.7 |
|  | 90870076 | -6.7 |
|  | 11131877 | -6.6 |
|  | 11624798 | -6.6 |
|  | 12116585 | -6.6 |
|  | 132210512 | -6.6 |
|  | 132212629 | -6.6 |
|  | 1777572 | -6.6 |
|  | 44394021 | -6.6 |
|  | 44437436 | -6.6 |
|  | 44575263 | -6.6 |
|  | 46907273 | -6.6 |
|  | 52947253 | -6.6 |
|  | 56776171 | -6.6 |
|  | 57403993 | -6.6 |
|  | 59741075 | -6.6 |
|  | 59897192 | -6.6 |
|  | 59897195 | -6.6 |
|  | 68103289 | -6.6 |
|  | 90796586 | -6.6 |
|  | 11717280 | -6.5 |
|  | 59876515 | -6.5 |
|  | 59921242 | -6.5 |
|  | 6373011 | -6.5 |
|  | 90676608 | -6.5 |
|  | 91034308 | -6.5 |
|  | 117673341 | -6.4 |
|  | 129010368 | -6.4 |
|  | 38350563 | -6.4 |
|  | 44393932 | -6.4 |
|  | 52950159 | -6.4 |
|  | 123197430 | -6.3 |
|  | 44393868 | -6.3 |
|  | 44575277 | -6.3 |
|  | 59881735 | -6.3 |
|  | 68402469 | -6.3 |
|  | 118122636 | -6.2 |
|  | 118712679 | -6.2 |
|  | 21679044 | -6.2 |
|  | 44393828 | -6.2 |
|  | 44393895 | -6.2 |
|  | 59881734 | -6.2 |
|  | 59897190 | -6.2 |
|  | 59897197 | -6.2 |
|  | 91066252 | -6.2 |
|  | 91358749 | -6.2 |
|  | 118712682 | -6.1 |
|  | 123377869 | -6.1 |
|  | 44393882 | -6.1 |
|  | 59881738 | -6.1 |
|  | 59897200 | -6.1 |
|  | 90718614 | -6.1 |
|  | 44393935 | -6 |
|  | 73265376 | -6 |
|  | 90985283 | -6 |
|  | 59876522 | -5.9 |

**Supplementary Table S5.** Molecular docking analysis results of all screened 22 analogs from virtual screening along with AGP, aspirin, and rofecoxib.

| **S. No.** | **Analogs PubChem ID** | **Binding Energy (kcal/mol)** |
| --- | --- | --- |
|  | Rofecoxib | -8.17 |
|  | Aspirin | -5.61 |
|  | AGP | -7.95 |
|  | 132210508 | -9.35 |
|  | 132210370 | -8.92 |
|  | 132210409 | -8.56 |
|  | 132210478 | -8.55 |
|  | 132210549 | -8.31 |
|  | 59876521 | -8.11 |
|  | 59876529 | -8.1 |
|  | 132210388 | -7.98 |
|  | 44574537 | -7.96 |
|  | 46871816 | -7.9 |
|  | 71550939 | -7.88 |
|  | 127040428 | -7.54 |
|  | 127042992 | -7.5 |
|  | 129317078 | -7.3 |
|  | 16122395 | -6.77 |
|  | 132210515 | -6.3 |
|  | 132210396 | -5.7 |
|  | 132210372 | -5.53 |
|  | 10041596 | -5.4 |
|  | 44409328 | -5.3 |
|  | 59876523 | -4.52 |
|  | 132217538 | -3.2 |

**Supplementary Table S6.** Statistical analysis of MD simulation results in term of RMSD, RMSF, and number of Hbonds.

| **Name** | **RMSD (nm)**  **Backbone after lsq fit to backbone** | | | **RMSF (nm)** | | | **Number of Hbonds** | |
| --- | --- | --- | --- | --- | --- | --- | --- | --- |
|  | **Min** | **Max** | **Average** | **Min** | **Max** | **Average** | **Protein (Max)** | **Protein-ligand (Max)** |
| Protein | 0.0000143 | 0.75 | 0.64 ± 0.10 | 0.06 | 1.21 | 0.20 ± 0.13 | 514 | - |
| Rofecoxib | 0.0000017 | 0.66 | 0.57 ± 0.06 | 0.06 | 1.42 | 0.18 ± 0.16 | 506 | 10 |
| Aspirin | 0.0000018 | 0.55 | 0.46 ± 0.05 | 0.06 | 1.02 | 0.19 ± 0.11 | 510 | 6 |
| AGP | 0.0000017 | 0.63 | 0.46 ± 0.08 | 0.06 | 0.85 | 0.20 ± 0.12 | 503 | 11 |
| A1 | 0.0000012 | 0.59 | 0.47 ± 0.07 | 0.06 | 1.08 | 0.18 ± 0.13 | 499 | 10 |
| A2 | 0.0000025 | 0.54 | 0.44 ± 0.05 | 0.06 | 1.25 | 0.19 ± 0.12 | 504 | 9 |
| A3 | 0.0000017 | 0.46 | 0.37 ± 0.03 | 0.06 | 0.90 | 0.18 ± 0.09 | 525 | 11 |
| A4 | 0.0000017 | 0.60 | 0.43 ± 0.08 | 0.06 | 1.66 | 0.21 ± 0.17 | 491 | 10 |
| A5 | 0.0003959 | 0.73 | 0.59 ± 0.13 | 0.06 | 1.80 | 0.21 ± 0.18 | 515 | 9 |
| A6 | 0.0000012 | 0.57 | 0.49 ± 0.03 | 0.06 | 0.74 | 0.18 ± 0.09 | 519 | 7 |
| A7 | 0.0006236 | 0.59 | 0.47 ± 0.04 | 0.50 | 0.94 | 0.16 ± 0.10 | 511 | 6 |
| Footnote: RMSD = Root Mean Square Deviation, RMSF = Root Mean Square Fluctuation, H bond = Hydrogen bond | | | | | | | | |

**Supplementary Table S7.** Important interacting amino acid residues with their RMSF value (nm).

| **_Residues_** | **_Protein_** | **_Rofecoxib_** | **_Aspirin_** | **_AGP_** | **_A1_** | **_A2_** | **_A3_** | **_A4_** | **_A5_** | **_A6_** | **_A7_** |
| --- | --- | --- | --- | --- | --- | --- | --- | --- | --- | --- | --- |
| _Val102_ | _0.17_ | _0.12_ | _0.14_ | _0.18_ | _0.16_ | _0.15_ | _0.16_ | _0.17_ | _0.14_ | _0.14_ | _0.13_ |
| _Arg106_ | _0.25_ | _0.14_ | _0.18_ | _0.21_ | _0.16_ | _0.22_ | _0.18_ | _0.2_ | _0.15_ | _0.13_ | _0.21_ |
| _Ile331_ | _0.14_ | _0.09_ | _0.14_ | _0.14_ | _0.13_ | _0.11_ | _0.13_ | _0.14_ | _0.13_ | _0.12_ | _0.12_ |
| _Val335_ | _0.11_ | _0.09_ | _0.13_ | _0.13_ | _0.10_ | _0.09_ | _0.11_ | _0.12_ | _0.09_ | _0.11_ | _0.10_ |
| _Leu338_ | _0.12_ | _0.12_ | _0.12_ | _0.18_ | _0.12_ | _0.10_ | _0.11_ | _0.15_ | _0.13_ | _0.12_ | _0.12_ |
| _Tyr341_ | _0.18_ | _0.18_ | _0.18_ | _0.26_ | _0.16_ | _0.13_ | _0.15_ | _0.18_ | _0.13_ | _0.14_ | _0.21_ |
| _Leu345_ | _0.14_ | _0.13_ | _0.16_ | _0.15_ | _0.18_ | _0.12_ | _0.11_ | _0.15_ | _0.17_ | _0.14_ | _0.14_ |
| _Ser339_ | _0.12_ | _0.22_ | _0.19_ | _0.21_ | _0.11_ | _0.10_ | _0.12_ | _0.14_ | _0.12_ | _0.15_ | _0.10_ |
| _Tyr371_ | _0.12_ | _0.11_ | _0.09_ | _0.13_ | _0.12_ | _0.10_ | _0.11_ | _0.18_ | _0.12_ | _0.13_ | _0.11_ |
| _Trp373_ | _0.12_ | _0.11_ | _0.09_ | _0.13_ | _0.12_ | _0.10_ | _0.11_ | _0.18_ | _0.12_ | _0.13_ | _0.11_ |
| _Arg499_ | _0.17_ | _0.22_ | _0.16_ | _0.18_ | _0.2_ | _0.15_ | _0.2_ | _0.18_ | _0.18_ | _0.13_ | _0.20_ |
| _Phe504_ | _0.11_ | _0.13_ | _0.14_ | _0.18_ | _0.18_ | _0.14_ | _0.18_ | _0.17_ | _0.14_ | _0.1_ | _0.26_ |
| _Met508_ | _0.14_ | _0.13_ | _0.10_ | _0.11_ | _0.11_ | _0.18_ | _0.18_ | _0.12_ | _0.12_ | _0.15_ | _0.12_ |
| _Val509_ | _0.18_ | _0.09_ | _0.10_ | _0.13_ | _0.13_ | _0.13_ | _0.13_ | _0.13_ | _0.11_ | _0.11_ | _0.11_ |
| _Gly512_ | _0.15_ | _0.11_ | _0.08_ | _0.14_ | _0.1_ | _0.09_ | _0.11_ | _0.12_ | _0.09_ | _0.09_ | _0.08_ |
| _Ala513_ | _0.11_ | _0.11_ | _0.10_ | _0.17_ | _0.11_ | _0.09_ | _0.12_ | _0.13_ | _0.11_ | _0.10_ | _0.09_ |
| _Ser516_ | _0.11_ | _0.09_ | _0.10_ | _0.12_ | _0.11_ | _0.10_ | _0.11_ | _0.12_ | _0.11_ | _0.11_ | _0.08_ |
| _Leu517_ | _0.13_ | _0.11_ | _0.11_ | _0.14_ | _0.14_ | _0.13_ | _0.14_ | _0.14_ | _0.12_ | _0.13_ | _0.11_ |

**Supplementary Table S8.** Statistical analysis of MD simulation results in term of Rg and average area per residue.

| **Name** | **Rg (nm)** | | | **Average area per residue (nm^2^)** | | |
| --- | --- | --- | --- | --- | --- | --- |
|  | **Min** | **Max** | **Average** | **Min** | **Max** | **Average** |
| Protein | 2.43 | 2.61 | 2.47 ± 0.02 | 0.001 | 2.05 | 0.45 ± 0.44 |
| Rofecoxib | 2.43 | 2.61 | 2.48 ± 0.03 | 0 | 2.21 | 0.45 ± 0.43 |
| Aspirin | 2.43 | 2.61 | 2.47 ± 0.02 | 0.003 | 2.06 | 0.46 ± 0.44 |
| AGP | 2.49 | 2.63 | 2.53 ± 0.01 | 0.001 | 2.06 | 0.47 ± 0.44 |
| A1 | 2.46 | 2.61 | 2.51 ± 0.02 | 0.001 | 2.11 | 0.46 ± 0.44 |
| A2 | 2.49 | 2.61 | 2.53 ± 0.01 | 0 | 2.18 | 0.46 ± 0.44 |
| A3 | 2.44 | 2.58 | 2.47 ± 0.01 | 0.001 | 2.27 | 0.47 ± 0.45 |
| A4 | 2.47 | 2.62 | 2.52 ± 0.02 | 0.001 | 2.49 | 0.46 ± 0.44 |
| A5 | 2.43 | 2.59 | 2.48 ± 0.03 | 0.002 | 2.44 | 0.46 ± 0.44 |
| A6 | 2.43 | 2.64 | 2.47 ± 0.03 | 0.004 | 2.22 | 0.46 ± 0.43 |
| A7 | 2.47 | 2.62 | 2.51 ± 0.01 | 0 | 2.19 | 0.46 ± 0.45 |

**Supplementary Table S9.** Statistical analysis of MD simulation results in term of SAS area, SAS volume, and SAS density.

| **Name** | **SAS Area (nm^2^)** | | | **SAS_volume (nm\s3\n)** | | | **SAS density (g/l)** | | |
| --- | --- | --- | --- | --- | --- | --- | --- | --- | --- |
|  | **Min** | **Max** | **Average** | **Min** | **Max** | **Average** | **Min** | **Max** | **Average** |
| Protein | 251.82 | 319.75 | 273.57 ± 12.36 | 114.65 | 124.35 | 118.36 ± 1.40 | 921.24 | 999.24 | 968.03 ± 11.44 |
| Rofecoxib | 256.77 | 320.01 | 273.13 ± 10.08 | 115.28 | 124.16 | 118.57 ± 1.18 | 922.65 | 993.72 | 966.30 ± 9.55 |
| Aspirin | 260.35 | 327.42 | 279.12 ± 9.10 | 115.28 | 124.88 | 119.06 ± 1.15 | 917.33 | 993.77 | 962.27 ± 9.31 |
| AGP | 265.18 | 327.25 | 284.44 ± 7.00 | 115.99 | 125.65 | 119.23 ± 1.08 | 911.74 | 987.68 | 960.76 ± 8.73 |
| A1 | 260.29 | 325.09 | 278.36 ± 8.82 | 115.42 | 125.31 | 118.83 ± 1.19 | 914.19 | 992.53 | 964.13 ± 9.63 |
| A2 | 263.75 | 314.93 | 280.64 ± 7.02 | 115.40 | 123.75 | 119.02 ± 1.05 | 925.71 | 992.73 | 962.61 ± 8.53 |
| A3 | 268.85 | 319.10 | 284.59 ± 6.61 | 115.37 | 123.82 | 119.24 ± 1.10 | 925.24 | 992.93 | 960.63 ± 8.90 |
| A4 | 263.24 | 327.75 | 281.85 ± 8.68 | 115.11 | 125.73 | 119.19 ± 1.15 | 911.19 | 995.19 | 961.21 ± 9.25 |
| A5 | 261.63 | 323.41 | 280.15 ± 8.36 | 115.05 | 124.44 | 119.12 ± 1.17 | 920.64 | 995.70 | 961.82 ± 9.49 |
| A6 | 256.80 | 329.83 | 278.00 ± 12.27 | 115.24 | 125.39 | 118.77 ± 1.43 | 913.62 | 994.13 | 964.69 ± 11.55 |
| A7 | 263.09 | 313.61 | 278.77 ± 6.70 | 115.19 | 124.62 | 118.50 ± 1.07 | 919.27 | 994.56 | 966.85± 8.71 |

**Supplementary Table S10.** Number of total interacting amino acids before and after MD simulation of aspirin, rofecoxib, AGP, and hit AGP analogs A1-7 with AF-COX-2 protein.

| **Compounds Name** | **Interacting amino acids before MD simulation** | **Interacting amino acids after MD simulation** |
| --- | --- | --- |
| **Aspirin** | Val335, *Leu338,* Ser339, Trp373, Phe504, *Met508, Val509,* Gly512, Ala513, **Ser516** | His75, Leu78, Val102, **Arg106,** Val335, Leu338, Ser339, Tyr341, **Arg499,** Phe504, *Val509,* *Ala513,* Leu517 |
| **Rofecoxib** | His75, Leu78, Met99, *Val102,* Arg106, Ile331, *Val335,* Tyr341, *Leu345,* Leu338, Ser339, **Arg499**, Phe504, *Val509,* *Ala513, Leu517* | Phe184, Ala188, Gln189, Phe191, Thr192, Phe195, Tyr334, *Leu338,* *Tyr371,* Leu376, *Trp373,* Val420, Ile503, Phe504, Met508, Ser516 |
| **AGP** | Leu78, Met99, *Val102, Arg106, Val335,* Leu338, Ser339, *Tyr341, Leu345,* Arg499, *Phe504,* **Met508, Val509,** Gly512, Ala513, **Ser516** | Pro71, Val74, His75, Leu78, Met99, *Val102,* Arg106, *Val335,* Leu338, Ser339, Tyr341, *Leu345,* Arg499, *Phe504*, *Val509,* *Ala513*, Leu517 |
| **A1** | Pro71, Val74, His75, Leu78, **Arg106**, *Val335,* *Leu338,* Ser339, Tyr341*, Leu345,* Trp373, Arg499, *Phe504,* Met508, **Val509,** **Glu510,** Gly512, *Ala513,* Ser516, Leu517 | Val74, Leu78, Met99, *Val102,* Ser105, **Arg106,** *Val335,* *Leu338,* Ser339, *Tyr341,* *Leu345,* Trp373, *Phe504,* Met508, **Val509,** Gly512, *Ala513,* Ser516, Leu517 |
| **A2** | *Val102,* **Arg106**, *Val335,* Leu338, Ser339, *Tyr341,* *Leu345,* Tyr371, Trp373, Arg499, *Phe504,* **Met508,** *Val509,* Gly512, **Ala513,** **Ser516,** Leu517 | Pro71, Val74, His75, Leu78, *Val102,* *Arg106,* *Val335,* *Leu338,* Ser339, *Tyr341,* *Leu345,* Trp373, Arg499, Phe504, *Val509,* *Ala513,* Ser516, *Leu517* |
| **A3** | Met99, *Val102,* Arg106, *Val335*, Leu338, Ser339, *Tyr341, Leu345,* **Tyr371,** Trp373, Arg499, *Phe504,* Met508, *Val509,* Gly512, *Ala513,* Ser516, Leu517 | Val74, Leu78, Met99, *Val102,* Ser105, **Arg106,** *Val335,* Leu338, Ser339, *Tyr341,* *Leu345,* *Phe504,* *Val509,* *Ala513,* Ser516, *Leu517* |
| **A4** | Met99, *Val102,* Arg106, *Val335,* Leu338, Ser339, *Tyr341,* *Leu345,* Trp373, Arg499, *Phe504,* **Met508,** *Val509,* Gly512, **Ala513,** **Ser516,** Leu517 | Pro71, Val74, His75, Leu78, *Val102,* Arg106, *Val335,* Leu338, Ser339, *Tyr341,* *Leu345,* **Arg499,** Phe504, *Val509,* Glu510, *Ala513,* Ser516, *Leu517* |
| **A5** | Val74, His75, Leu78, **Arg106,** *Val335, Leu338,* Ser339, Tyr341, *Leu345,* Trp373, **Arg499**, Phe504, **Met508,** *Val509,* Glu510, Gly512, **Ala513,** Ser516, *Leu517* | His75, Met99, *Val102,* Leu103, *Arg106,* *Val335,* Leu338, Ser339, *Tyr341,* *Leu345,* Trp373, Arg499, *Phe504,* Met508, **Val509,** Gly512, Ala513, **Ser516,** *Leu517* |
| **A6** | *Leu78,* Val102, Arg106, Tyr334, *Val335,* *Leu338,* Ser339, *Tyr341,* *Leu345,* Tyr371, Trp373, *Phe504,* Met508, *Val509,* Gly512, *Ala513,* Ser516, Leu517 | Val74, Leu78, Val102, Ser105, Arg106, *Val335,* Leu338, Ser339, **Tyr341,** Leu345, Trp373, Arg499, *Phe504,* Met508, *Val509,* Gly512, *Ala513,* Leu517 |
| **A7** | *Leu78,* Val102, Arg106, Tyr334, *Val335,* *Leu338,* Ser339, *Tyr341,* Leu345, Tyr371, Trp373, *Phe504,* *Val509,* Gly512, *Ala513,* Ser516, Leu517 | Lys68, Pro71, *Val74,* *Leu78,* *Val102,* Ser105, **Arg106,** Leu109, Ser339, Tyr341, Leu345, Phe456, Met457, Arg499, Val509, Glu510 |
| Footnote: Bold letter = Residues involved in the H-bond formation, Italics letter = Residues involved in the hydrophobic interactions | | |


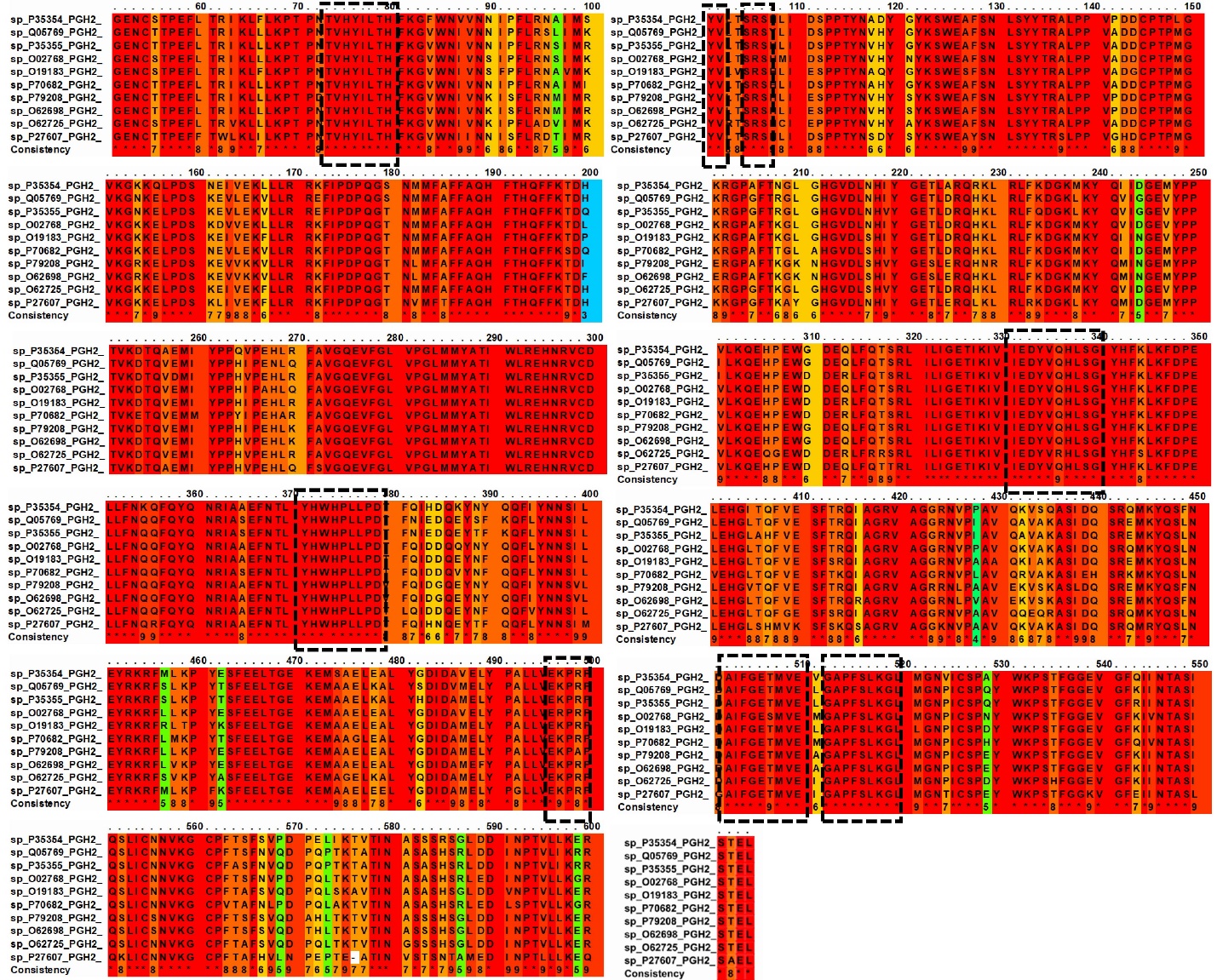


**
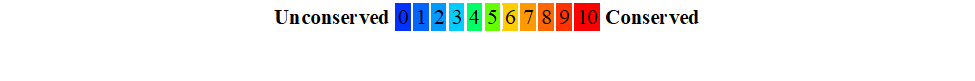
**

**Supplementary Figure S1.** Multiple sequence alignment of human COX-2 protein with other 9 mammal species indicating the conserved residues important for the ligand binding. This figure is plotted by using PRALINE program ^1^.

**
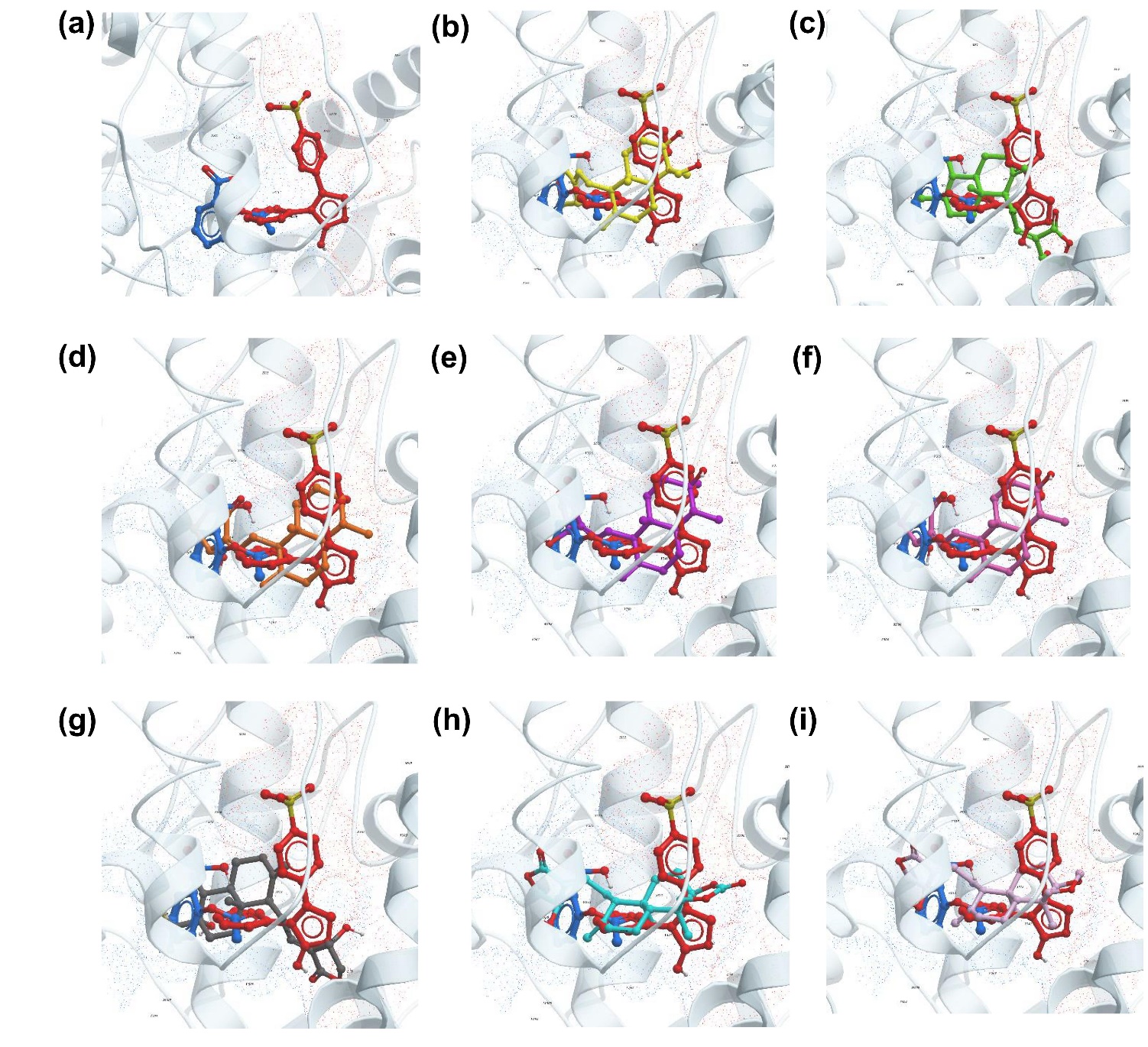
**

**Supplementary Figure S2.** 3D**-**spatial binding orientation of the reference drugs (aspirin and rofecoxib) and AGP analogs (AGP and A1-A7) in the ligand binding pocket of AF-COX-2. (a) the spatial orientation of aspirin (blue) and rofecoxib (red) in its binding pocket of the AF-COX-2 protein. (b)- (i) overlay of AGP and A1 to A7, respectively, with aspirin and rofecoxib complex.


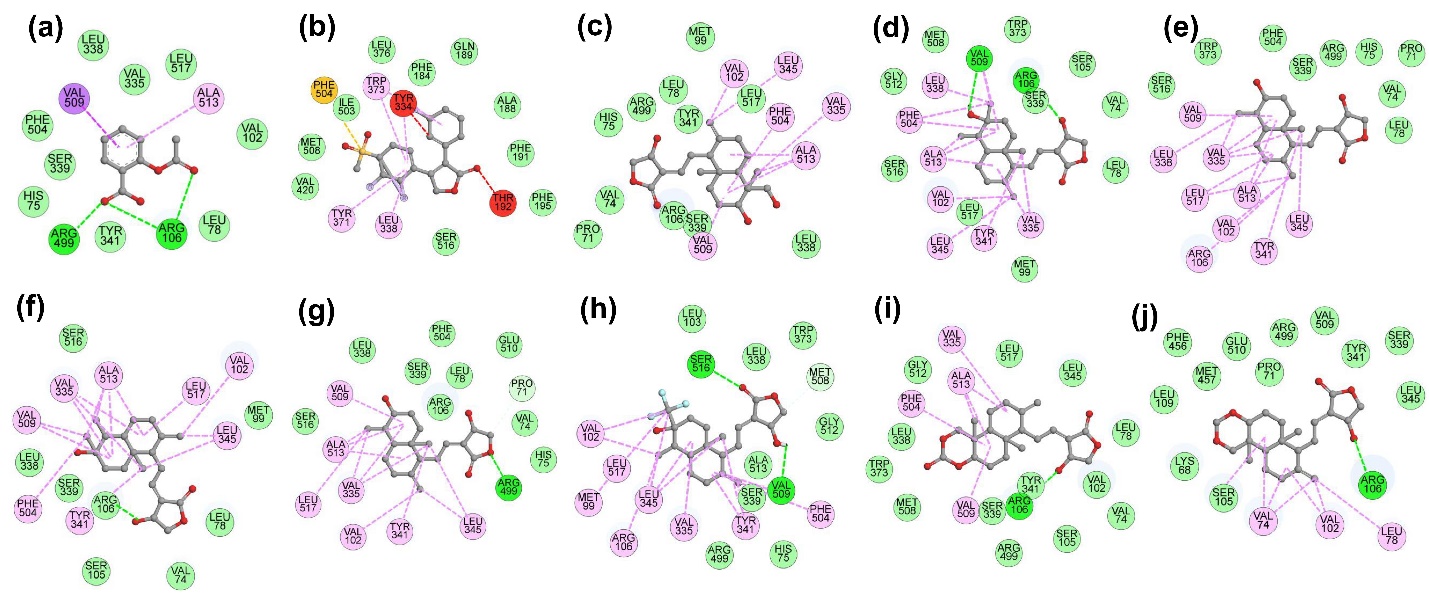


**Supplementary Figure S3.** After simulation 2D binding interactions of aspirin (a), rofecoxib (b), AGP (c), and AGP analogs (A1-A7) (d-j) with the binding site of AF-COX-2 protein. All the chemical compounds are depicted as ball and stick model; amino acids with their number are represented as a circle with three-letter code and a dashed line shows the interaction site of chemical compounds with amino acids. Color code: green = H-bonds, pink = HYD interactions.

**Reference:**

1. Simossis, V. A. & Heringa, J. PRALINE: A multiple sequence alignment toolbox that integrates homology-extended and secondary structure information. *Nucleic Acids Res.* **33**, 289–294 (2005).
